# Supplementary material for: Modeling habitat distribution and niche overlap of Asian horseshoe crabs: Implications for conservation
Source: PLoS One. 2025 May 28;20(5):e0324471. doi: 10.1371/journal.pone.0324471 (PMC12118861; doi:10.1371/journal.pone.0324471)
Supplement: Attachment — Submitted filename: PONE-D-25-08646_reviewer.pdf [file pone.0324471.s004.pdf]

PLOS ONE

Exploring habitat characteristics, distribution, and niche relationships of Asian horseshoe crabs: preserving living fossils and biodiversity

--Manuscript Draft--

**Manuscript Number:**	PONE-D-25-08646
**Article Type:**	Research Article
**Full Title:**	Exploring habitat characteristics, distribution, and niche relationships of Asian horseshoe crabs: preserving living fossils and biodiversity
**Short Title:**	Habitat characteristics, distribution, and niche relationships of Asian horseshoe crabs
**Corresponding Author:**	Zhong-Duo Wang<br>Guangdong Ocean University<br>zhanjiang, CHINA
**Keywords:**	Tachypleus tridentatus, Tachypleus gigas, Carcinoscorpius rotundicauda, Potential habitats, Niche overlap, Biodiversity
**Abstract:**	Horseshoe crabs, recognized as "living fossils" in evolutionary history, have consistently been a focal point of biological research. However, information regarding their habitat attributes, patterns, and niche relationships, particularly for Asian horseshoe crabs, remains incomplete. This study aims to comprehensively analyze the habitat characteristics, potential habitat distributions, niche overlaps and similarities, as well as the habitat patterns and refugia locations during the Last Glacial Maximum (LGM) of three Asian horseshoe crab species: Tachypleus tridentatus, Carcinoscorpius rotundicauda, and Tachypleus gigas. By employing a combination of MaxEnt Modeling and niche analysis, we have identified water depth and distance to land as the common key factors influencing the habitat distribution of these three species. Additionally, different species are affected by other specific environmental factors. For example, the distribution of T. tridentatus is constrained by the summer (May-Oct) maximum chlorophyll-a, the habitat of C. rotundicauda is closely related to the minimum chlorophyll-a, and habitat selection for T. gigas is significantly affected by wind speed. In terms of niche overlap, the highest degree of overlap is observed between C. rotundicauda and T. gigas, while the overlap between T. tridentatus and T. gigas is the lowest. During the LGM, the refugia of T. tridentatus were primarily located at the estuaries of the Minjiang and Pearl Rivers, whereas the refugia of both C. rotundicauda and T. gigas were situated near the Strait of Malacca. This study provides a scientific basis for formulating effective conservation and management strategies and holds significant importance for protecting these precious "living fossils" and maintaining biodiversity.
**Order of Authors:**	Jian Liao
	Chun-Hui Xiong
	Gao-Cong Li
	Jia-Yu Li
	Yuan-Feng Yang
	Shui-Yuan Zhang
	Yi-Yang Li
	Kai-Lin Zeng
	Mei-Ling Hu
	Yu-Song Guo
	Zhong-Duo Wang
**Additional Information:**	
**Question**	**Response**

Powered by Editorial Manager® and ProduXion Manager® from Aries Systems Corporation

**Financial Disclosure**<br><br>Enter a financial disclosure statement that describes the sources of funding for the work included in this submission. Review the submission guidelines for detailed requirements. View published research articles from *PLOS ONE* for specific examples.<br><br>This statement is required for submission and **will appear in the published article** if the submission is accepted. Please make sure it is accurate.<br><br>**Funded studies**<br>Enter a statement with the following details:<br>• Initials of the authors who received each award<br>• Grant numbers awarded to each author<br>• The full name of each funder<br>• URL of each funder website<br>• Did the sponsors or funders play any role in the study design, data collection and analysis, decision to publish, or preparation of the manuscript?<br><br>Did you receive funding for this work?	Yes
Please add funding details.<br>as follow-up to "**Financial Disclosure**<br><br>Enter a financial disclosure statement that describes the sources of funding for the work included in this submission. Review the submission guidelines for detailed requirements. View published research articles from *PLOS ONE* for specific examples.<br><br>This statement is required for submission and **will appear in the published article** if the submission is accepted. Please make sure it is accurate.	This study was funded by the Guangdong Provincial Field Observation and Research Station for Marine Ecosystem in Hanjiang River Estuary - Nanao Island Area Open Fund (Grant No. HNS202407), the National Key Research and Development Program of China (Grant No. 2024YFD2401803 & 2024YFD2401802), the Guangdong Provincial Ordinary University Youth Innovative Talent Project in 2024 (Grant No. 2024KQNCX134), the Guangdong Provincial Special Fund Project for Talent Development Strategy in 2024 (Grant No. 2024R3005), and the Guangdong Ocean University Scientific Research Startup Funding Project (Grant No. 060302022312).

Powered by Editorial Manager® and ProduXion Manager® from Aries Systems Corporation

**Funded studies**<br>Enter a statement with the following details:<br>• Initials of the authors who received each award<br>• Grant numbers awarded to each author<br>• The full name of each funder<br>• URL of each funder website<br>• Did the sponsors or funders play any role in the study design, data collection and analysis, decision to publish, or preparation of the manuscript?<br><br>Did you receive funding for this work?"	
Please select the country of your main research funder (please select carefully as in some cases this is used in fee calculation).<br>as follow-up to "**Financial Disclosure**<br><br>Enter a financial disclosure statement that describes the sources of funding for the work included in this submission. Review the submission guidelines for detailed requirements. View published research articles from *PLOS ONE* for specific examples.<br><br>This statement is required for submission and **will appear in the published article** if the submission is accepted. Please make sure it is accurate.<br><br>**Funded studies**<br>Enter a statement with the following details:<br>• Initials of the authors who received each award<br>• Grant numbers awarded to each author<br>• The full name of each funder<br>• URL of each funder website<br>• Did the sponsors or funders play any role in the study design, data collection and analysis, decision to publish, or preparation of the manuscript?<br><br>Did you receive funding for this work?"	CHINA - CN
**Competing Interests**	The authors have declared that no competing interests exist.

Use the instructions below to enter a competing interest statement for this submission. On behalf of all authors, disclose any competing interests that could be perceived to bias this work—acknowledging all financial support and any other relevant financial or non-financial competing interests.<br><br>This statement is required for submission and **will appear in the published article** if the submission is accepted. Please make sure it is accurate and that any funding sources listed in your Funding Information later in the submission form are also declared in your Financial Disclosure statement.<br><br>View published research articles from *PLOS ONE* for specific examples.<br><br>**NO authors have competing interests**<br><br>Enter: *The authors have declared that no competing interests exist.*<br><br>**Authors with competing interests**<br><br>Enter competing interest details beginning with this statement:<br><br>*I have read the journal's policy and the authors of this manuscript have the following competing interests: [insert competing interests here]*<br><br>* typeset	
**Ethics Statement**<br><br>Enter an ethics statement for this submission. This statement is required if the study involved:<br><br>• Human participants<br>• Human specimens or tissue<br>• Vertebrate animals or cephalopods<br>• Vertebrate embryos or tissues	N/A

• Field research<br><br>Write "N/A" if the submission does not require an ethics statement.<br><br>General guidance is provided below. Consult the submission guidelines for detailed instructions. **Make sure that all information entered here is included in the Methods section of the manuscript.**	

Powered by Editorial Manager® and ProduXion Manager® from Aries Systems Corporation

**Format for specific study types**<br><br>**Human Subject Research (involving human participants and/or tissue)**<br>• Give the name of the institutional review board or ethics committee that approved the study<br>• Include the approval number and/or a statement indicating approval of this research<br>• Indicate the form of consent obtained (written/oral) or the reason that consent was not obtained (e.g. the data were analyzed anonymously)<br><br>**Animal Research (involving vertebrate animals, embryos or tissues)**<br>• Provide the name of the Institutional Animal Care and Use Committee (IACUC) or other relevant ethics board that reviewed the study protocol, and indicate whether they approved this research or granted a formal waiver of ethical approval<br>• Include an approval number if one was obtained<br>• If the study involved *non-human primates*, add *additional details* about animal welfare and steps taken to ameliorate suffering<br>• If anesthesia, euthanasia, or any kind of animal sacrifice is part of the study, include briefly which substances and/or methods were applied<br><br>**Field Research**<br><br>Include the following details if this study involves the collection of plant, animal, or other materials from a natural setting:<br>• Field permit number<br>• Name of the institution or relevant body that granted permission	
**Data Availability**<br><br>Authors are required to make all data underlying the findings described fully available, without restriction, and from the time of publication. PLOS allows rare exceptions to address legal and ethical concerns. See the PLOS Data Policy and FAQ for detailed information.	Yes - all data are fully available without restriction

Powered by Editorial Manager® and ProduXion Manager® from Aries Systems Corporation

A Data Availability Statement describing where the data can be found is required at submission. Your answers to this question constitute the Data Availability Statement and **will be published in the article**, if accepted.<br><br>**Important:** Stating 'data available on request from the author' is not sufficient. If your data are only available upon request, select 'No' for the first question and explain your exceptional situation in the text box.<br><br>Do the authors confirm that all data underlying the findings described in their manuscript are fully available without restriction?	
**Describe where the data may be found in full sentences. If you are copying our sample text, replace any instances of XXX with the appropriate details.**<br><br>• If the data are **held or will be held in a public repository**, include URLs, accession numbers or DOIs. If this information will only be available after acceptance, indicate this by ticking the box below. For example: *All XXX files are available from the XXX database (accession number(s) XXX, XXX.).*<br>• If the data are all contained **within the manuscript and/or Supporting Information files**, enter the following: *All relevant data are within the manuscript and its Supporting Information files.*<br>• If neither of these applies but you are able to provide **details of access elsewhere**, with or without limitations, please do so. For example:<br><br>*Data cannot be shared publicly because of [XXX]. Data are available from the XXX Institutional Data Access / Ethics Committee (contact via XXX) for researchers who meet the criteria for access to confidential data.*<br><br>*The data underlying the results presented in the study are available from (include the name of the third party*	All relevant data are within the manuscript and its Supporting Information files.

and contact information or URL).<br>• This text is appropriate if the data are owned by a third party and authors do not have permission to share the data.<br><br>* typeset	
Additional data availability information:	Tick here if your circumstances are not covered by the questions above and you need the journal’s help to make your data available.

Powered by Editorial Manager® and ProduXion Manager® from Aries Systems Corporation

Manuscript

Click here to access/download;Manuscript;Jian Liao20250222.docx

Exploring habitat characteristics, distribution, and niche relationships of Asian horseshoe crabs: preserving living fossils and biodiversity

Jian Liao[1], Chun-Hui Xiong[2], Gao-Cong Li[3], Jia-Yu Li[1], Yuan-Feng Yang[1], Shui-Yuan Zhang[1], Yi-Yang Li[1], Kai-Lin Zeng[1], Mei-Ling Hu[1], Yu-Song Guo[1], Zhong-Duo Wang[1,4*]

1. Key Laboratory of Aquaculture in South China Sea for Aquatic Economic Animals of Guangdong Higher Education Institutes, college of fisheries, Guangdong Ocean University, Zhanjiang, 524088, China;

2. Guangdong Provincial Field Observation and Research Station for Marine Ecosystem in Hanjiang River Estuary - Nanao Island Area, Guangzhou, 510300, China.

3. College of Electronics and Information Engineering, Guangdong Ocean University, Zhanjiang, 524088, China;

4. Guangdong Provincial Key Laboratory of Aquatic Animal Disease control and Healthy culture, college of fisheries, Guangdong Ocean University, Zhanjiang, 524088, China

* Corresponding author. E-mail address: wangzd@gdou.edu.cn (ZD. Wang)

ABSTRACT

Horseshoe crabs, recognized as "living fossils" in evolutionary history, have consistently been a focal point of biological research. However, information regarding their habitat attributes, patterns, and niche relationships, particularly for Asian horseshoe crabs, remains incomplete. This study aims to comprehensively analyze the habitat characteristics, potential habitat distributions, niche overlaps and similarities, as well as the habitat patterns and refugia locations during the Last Glacial Maximum (LGM) of three Asian horseshoe crab species: *Tachypleus tridentatus*, *Carcinoscorpius rotundicauda*, and *Tachypleus gigas*. By employing a combination of MaxEnt Modeling and niche analysis, we have identified water depth and distance to land as the common key factors influencing the habitat distribution of these three species. Additionally, different species are affected by other specific environmental factors. For example, the

distribution of *T. tridentatus* is constrained by the summer (May-Oct) maximum chlorophyll-a, the habitat of *C. rotundicauda* is closely related to the minimum chlorophyll-a, and habitat selection for *T. gigas* is significantly affected by wind speed. In terms of niche overlap, the highest degree of overlap is observed between *C. rotundicauda* and *T. gigas*, while the overlap between *T. tridentatus* and *T. gigas* is the lowest. During the LGM, the refugia of *T. tridentatus* were primarily located at the estuaries of the Minjiang and Pearl Rivers, whereas the refugia of both *C. rotundicauda* and *T. gigas* were situated near the Strait of Malacca. This study provides a scientific basis for formulating effective conservation and management strategies and holds significant importance for protecting these precious "living fossils" and maintaining biodiversity.

Keywords: *Tachypleus tridentatus*, *Tachypleus gigas*, *Carcinoscorpius rotundicauda*, Potential habitats, Niche overlap, Biodiversity

1. Introduction

As living fossils, horseshoe crabs represent the sole extant members of the xiphosurans, and their morphology is believed to have remained unchanged for over 200 million years, making them an indispensable component of marine ecosystems (John et al., 2018; Rudkin and Young, 2009). With their unique biological characteristics and ecological functions, they play an irreplaceable role in maintaining marine ecological balance and promoting biodiversity (Rudkin and Young, 2009). These ancient creatures, whose fossils date back to the Ordovician period of the Palaeozoic Era, approximately 475 million years ago, have become hotspots in scientific research due to their unique blue blood, complex physiological mechanisms, and significant position in the history of biological evolution (Nong, 2021; Xiong et al., 2023; Zhu et al., 2020). Globally, there are four species of horseshoe crabs: American horseshoe crab *Limulus polyphemus*, Chinese horseshoe crab *Tachypleus tridentatus*, coastal horseshoe crab *Tachypleus gigas*, and mangrove horseshoe crab *Carcinoscorpius rotundicauda* (John et al., 2018; Sekiguchi and Sugita, 1980), and all of them have been listed in the IUCN Red List of Threatened Species (IUCN, 2024; Wang et al., 2019). Among them, *T. tridentatus*, *T. gigas*, and *C. rotundicauda* are primarily distributed in Asian seas and are therefore also referred to as

Asian horseshoe crabs (John et al., 2018). In contrast, *L. polyphemus* is distributed in the Americas and is thus called the American horseshoe crab (Smith et al., 2017). *T. tridentatus*, with its extensive geographical distribution in East Asia and important ecological functions, plays a crucial role in the marine food chain and is significant for maintaining ecological balance (John et al., 2018). *T. gigas* is widely distributed in tropical and subtropical seas of Southeast Asia, particularly in Vietnam, the Philippines, Indonesia, and other regions, often residing in intertidal sandy or muddy beaches (Sheikh et al., 2021). *C. rotundicauda* primarily inhabits the unique mangrove ecosystems of tropical seas and is highly dependent on these ecosystems (Fairuz-Fozi et al., 2021).

However, with the continuous expansion of human activities and the sustained deterioration of marine environmental quality, these ancient and precious Asian horseshoe crab populations are facing unprecedented survival crises (Carmichael et al., 2015; Rudkin and Young, 2009; Tanacredi et al., 2009). From shallow-sea fishing to deep-sea resource exploitation, human exploitation of marine resources has directly led to sharp declines in numerous marine species populations, including Asian horseshoe crabs (Carmichael et al., 2015; Ling et al., 2013). Overfishing not only reduces their direct populations but also disrupts the integrity of their food chains, affecting the natural recovery ability of the populations (Le Pape et al., 2017). Meanwhile, the habitats of Asian horseshoe crabs have also suffered severe damage (Wang et al., 2019). With the acceleration of urbanization, large amounts of beaches and wetlands, particularly nearshore spawning grounds, have been developed for construction, transforming these ideal habitats for Asian horseshoe crabs into inaccessible forbidden zones (Wang et al., 2019). Additionally, waste generated from industrialization and agricultural activities in coastal areas is directly discharged into the ocean without effective treatment, not only polluting water quality but also damaging the underwater ecological environment, further compressing the living space of Asian horseshoe crabs. Therefore, conducting in-depth research on the potential habitats of the three Asian horseshoe crab species (*T. tridentatus*, *T. gigas*, and *C. rotundicauda*) and the important ecological factors affecting their distribution is crucial for formulating scientific and reasonable conservation strategies, promoting population recovery, and protecting biodiversity.

Species distribution models are an effective method to help researchers gain a deeper

understanding of the intricate relationship between species and their environments (Ahmad et al., 2019; Zhao et al., 2024). Among them, the MaxEnt model based on the principle of maximum entropy integrates environmental variables and species distribution data to construct a probability model of species distribution (Elith et al., 2011; Phillips et al., 2006). The core advantage of this model lies in its strong predictive ability and efficient use of data (Liao et al., 2022). Compared to other models, MaxEnt has relatively relaxed requirements for data, and can make accurate predictions with only a small amount of species distribution data and environmental factor data (Liao et al., 2023). In addition, the MaxEnt model can also handle environmental factors with nonlinear relationships and fully consider the spatial heterogeneity of species distribution, thereby effectively predicting the potential distribution areas of species under different environmental conditions (Phillips et al., 2006; Pshegusov and Chadaeva, 2023). After more than a decade of development, the MaxEnt model, as the mainstream method in species distribution modeling, has been systematically and maturely applied in the field of terrestrial biology, and is entering a rapid development stage in the field of marine biology (Du et al., 2017; Stirling et al., 2016; Yi et al., 2016).

This study utilizes the MaxEnt model to predict the potential habitats of the three Asian horseshoe crab species, analyzes the key ecological factors influencing their distribution, and examines the niche overlap and similarity among them, providing strong support for formulating effective conservation and management strategies. The research results will help guide relevant departments and institutions in formulating scientific conservation plans, optimizing resource allocation, strengthening habitat protection, and promoting population recovery. Meanwhile, this study will also provide methods and ideas for the conservation research of other endangered species, promoting the development of global biodiversity conservation efforts.

2. Materials and Methods

2.1 Species occurrence sites

The distribution point data for *T. tridentatus*, *C. rotundicauda*, and *T. gigas* primarily originated from the Global Biodiversity Information Facility (GBIF, website: https://www.gbif.org/), the Ocean Biodiversity Information System (website: https://obis.org/),

and literature reviews. During the data preprocessing phase, we carefully cross-checked each occurrence record against the known distribution ranges of *T. tridentatus*, *C. rotundicauda*, and *T. gigas*, and eliminated duplicates or anomalous values that fell outside these established boundaries (Liao et al., 2023). To further reduce spatial autocorrelation and data redundancy, we adopted a grid system method, dividing the study area into 5km×5km grid cells. Within each grid cell, we retained only the occurrence point closest to the geometric center of the grid, thereby reducing the density of the dataset while preserving its key spatial distribution characteristics, effectively avoiding issues of overfitting or bias (Zhang et al., 2018). After rigorous screening and spatial analysis, we finally compiled datasets containing 268 coordinate records for *Tachypleus tridentatus*, 305 for *Carcinoscorpius rotundicauda*, and 231 for *Tachypleus gigas* (**Figure 1**).

2.2 Environmental variables

To analyze the distribution patterns of three Asian horseshoe crab species, *T. tridentatus*, *C. rotundicauda*, and *T. gigas*, we downloaded 48 marine environmental variables from the Global Marine Environment Dataset (GMED, https://gmed.auckland.ac.nz/index.html). These variables were categorized into 26 physical variables, 9 chemical variables, and 13 nutritional variables, as detailed in Table 1, with a resolution of 2.5 minutes. The raster layer encompassed data from both the current period and the Last Glacial Maximum (LGM). Subsequently, we utilized the "Extract Multi Values to Points" tool in the ArcGIS toolbox to extract the values of these environmental variables from the distribution points of the three Asian horseshoe crab species. Following that, in the R programming language, we employed the *usdm* package (version 1.1-18) to conduct Variance Inflation Factor (VIF) analysis, setting the VIF threshold at <10 to eliminate factors with multicollinearity, as recommended in the study by Naimi et al. (2014).

Table 1. Global marine environmental variable data (including physical variables, chemical variables, and nutrient variables) and the variance inflation factor (VIF) values of variables after screening through VIF analysis. Variables with VIF values greater than 10 were removed.

**Environment Variable**		***T. tridentatus***	***T. gigas***	***C. rotundicauda***	**Grid Layer Code**
Physical	Depth	**1.343**	**1.940**	**1.559**	gb_depth
	Slope	>10	>10	>10	gb_slope
	Aspect (East-West)	>10	>10	>10	msp_asp_ew
	Aspect (North-South)	>10	>10	>10	msp_asp_ns
	Distance to Land	**1.576**	**1.695**	**1.834**	gb_land_distance
	Distance to Port	>10	>10	>10	port_distance
	Ice Concentration Annual	>10	>10	>10	aq_ice_amn
	Ice Concentration (May-Oct)	>10	>10	>10	aq_ice_sum
	Ice Concentration (Nov-Apr)	>10	>10	>10	aq_ice_win
	Tide average	**1.480**	**1.671**	**1.191**	kg_tide_average
	Wave height	**9.496**	**5.693**	**6.564**	aq_waveheight
	Wind speed	**2.835**	**1.418**	**3.694**	kg_wind_speed
	Surface Current	**3.280**	**1.311**	**1.846**	ecco2_uv_surface_current
	Euphotic Layer Bottom Depth	>10	>10	>10	gc_zeu_mean
	Diffuse attenuation coefficient	>10	>10	>10	bo_da_mean
	Maximum Temperature	**7.383**	**4.509**	**5.475**	bo_sst_maximum
	Average Temperature	>10	**5.522**	>10	bo_sst_average
	Minimum Temperature	>10	>10	>10	bo_sst_minimum
	Range Temperature	>10	>10	>10	bo_sst_range
	Summer Temperature (May-Oct)	**1.597**	**1.859**	**1.595**	na_may_oct_sst
	Winter Temperature (Nov-Apr)	>10	>10	>10	na_nov_apr_sst
	Seabed Temperature	>10	>10	>10	kg_b_temp

	Water Column Temperature	>10	>10	>10	na_b_temp
	Salinity Mean	**5.859**	>10	**6.064**	bo_salinity
	Bottom Salinity	>10	>10	>10	na_b_salinity
	Photosynthetically Active Radiation	**8.304**	**5.918**	**4.183**	bo_parmean
Chemical	Average Chlorophyll-a	>10	>10	>10	bo_chla_average
	Maximum Chlorophyll-a	>10	>10	>10	bo_chla_maximum
	Minimum Chlorophyll-a	**2.648**	**2.594**	**3.637**	bo_chla_minimum
	Chlorophyll-a Range	**1.972**	**3.354**	**3.901**	bo_chla_range
	Summer (May-Oct) Maximum Chlorophyll-a	**1.875**	>10	**2.997**	kg_chla_summax
	Winter (Nov-Apr) Maximum Chlorophyll-a	**1.821**	**1.523**	>10	kg_chla_winmax
	Primary Productivity	**6.321**	>10	**6.627**	aq_primprod
	pH	**3.888**	>10	**6.599**	bo_ph
	Total Suspended Matter	>10	>10	>10	gc_tsm_mean
Nutrients	Calcite	>10	>10	>10	bo_calcite
	Nitrate	**7.558**	>10	>10	bo_nitrate
	Bottom Nitrate	>10	>10	>10	kg_b_nitrate
	Phosphate	>10	>10	>10	kg_phosphate
	Bottom Phosphate	>10	>10	>10	kg_b_phosphate
	Silicate	**8.628**	**2.437**	**3.916**	bo_silicate
	Bottom Silicate	**3.857**	**2.488**	>10	kg_b_silicate
	Dissolved Oxygen	**9.175**	**2.009**	**8.670**	bo_o2dis
	Bottom Dissolved Oxygen	**7.893**	**3.453**	**2.838**	kg_b_o2dissolve
	Saturated Oxygen	**7.529**	**3.203**	**1.475**	kg_s_o2saturate
	Bottom Utilized Oxygen	>10	>10	>10	kg_b_o2utilized
	Particulate Organic Carbon	>10	>10	>10	gc_poc_mean
	Particulate InOrganic Carbon	>10	>10	>10	gc_pic_mean

2.3 Potential Habitat and Niche Analysis

In this study, we employed the Maximum Entropy (MaxEnt) modeling approach, integrating distribution point data with a multitude of marine environmental variables (including physical, chemical, and nutritional factors), to predict the suitable habitat distribution of *T. tridentatus*, *C. rotundicauda*, and *T. gigas* using MaxEnt version 3.4.1 (Phillips et al., 2006). To ensure prediction accuracy, the process involved randomly selecting 25% of the distribution points as the test set, while the remaining 75% served as the training set for model calibration and testing. This process was repeated ten times to enhance reliability (Liao et al., 2022). The MaxEnt algorithm iterated until convergence, with a convergence threshold set at 0.00001. Model performance was independently evaluated using Receiver Operating Characteristic (ROC) curve analysis, and the Area Under the Curve (AUC) value was utilized as an indicator. The AUC categories were defined as excellent ($AUC > 0.9$), good ($0.8 \leqslant AUC < 0.9$), moderate ($0.7 \leqslant AUC < 0.8$), poor ($0.6 \leqslant AUC < 0.7$), and fail ($AUC < 0.6$) (Liao et al., 2023). Furthermore, the Jackknife Test was conducted to assess the contribution and importance of each environmental variable. The model output, in ASC format, was refined into raster layers using ArcGIS 10.8 for habitat suitability classification. Based on the suitability values derived from the simulation results, potential habitats were categorized into four classes: highly suitable habitat (suitability value > 0.6), moderately suitable habitat ($0.4 <$ suitability value $\leqslant 0.6$), low suitable habitat ($0.2 <$ suitability value $\leqslant 0.4$), and unsuitable habitat (suitability value $\leqslant 0.2$) (Liao et al., 2023, 2022). Specifically, refugia were defined as habitats with suitability values exceeding 0.8 during the Last Glacial Maximum (Liao et al., 2023).

To facilitate an in-depth analysis of the distribution patterns of the three Asian horseshoe crab species and accurately quantify the degree of niche overlap among them, the "*ecospat*" package was employed in R (Di Cola et al., 2017). Schoener's D index, ranging from 0 (indicating no overlap) to 1 (indicating complete overlap), was used to quantify niche overlap. Additionally, niche similarity between each pair of the three Asian horseshoe crab species was calculated using the "*ecospat*" package to understand and compare their positions and relationships within the ecological space (Di Cola et al., 2017). The null hypothesis for niche similarity typically assumes that the degree of overlap between two species in niche space is

random or solely determined by abiotic factors, without the influence of interspecific interactions.

3.Results

3.1 Assessment of model performance and identification of key environmental variables

After ROC curve analysis, the AUC values for the training and testing sets of the potential habitat prediction model for *T. tridentatus* were 0.9963 and 0.9958, respectively. For the model of *C. rotundicauda*, the AUC values for the training and testing sets were 0.9980 and 0.9972, respectively. For the model of *T. gigas*, the AUC values for the training and testing sets were 0.9974 and 0.9972, respectively. The AUC values for both the training and testing sets of all three Asian horseshoe crab species exceeded 0.9, indicating the reliability of the MaxEnt model in predicting the potential habitats of these species. Variance Inflation Factor (VIF) analysis was conducted on 48 marine environmental variables, which were categorized into 26 physical environmental factors, 9 chemical environmental factors, and 13 nutritional environmental factors. Following the VIF analysis, 21 environmental variables were retained for *T. tridentatus*, 19 for *C. rotundicauda*, and 21 for *T. gigas* for modeling and analysis. The VIF values for these selected variables are presented in **Table 1**. The results of the MaxEnt model analysis revealed the environmental variables that most significantly shape the current potential habitats of the three Asian horseshoe crab species. For *T. tridentatus*, the variables in descending order of importance were: water depth (29.75%), minimum chlorophyll-a concentration (23.98%), distance to land (13.49%), wave height (8.59%), and silicate (7.05%). For *C. rotundicauda*, the variables were: minimum chlorophyll-a concentration (55.85%), distance to land (14.28%), summer temperature (May-Oct) (11.17%), water depth (10.03%), and silicate (2.12%). Lastly, for *T. gigas*, the variables were: water depth (50.71%), nitrate (11.7747%), wind speed (11.6947%), summer temperature (May-Oct) (8.1283%), and distance to land (6.5095%). Further evaluation of the influence of environmental variables on the potential habitat distribution of three species of Asian horseshoe crabs was conducted using a jackknife test, enabling the identification of the five most significant environmental factors for each species. For *T. tridentatus*, the order of importance of environmental factors affecting its potential habitat was as follows: water depth > distance to land > maximum chlorophyll-a content during

summer > minimum chlorophyll-a content > bottom silicate (**Figure 2A**). For *C. rotundicauda*, the order of importance for its potential habitat distribution was: water depth > distance to land > minimum chlorophyll-a content > maximum chlorophyll-a content during summer > chlorophyll-a range (**Figure 2B**). Lastly, for *T. gigas*, the order of importance for its potential habitat distribution was: water depth > distance to land > wind speed > maximum temperature > maximum chlorophyll-a content (**Figure 2C**).

3.2 Potential habitats of *T. tridentatus*, *C. rotundicauda*, and *T. gigas*

The potential habitat distribution maps for *T. tridentatus*, *C. rotundicauda*, and *T. gigas*, as predicted by the model, detail the regions of highly suitable, moderately suitable, and lowly suitable habitats, as illustrated specifically in **Figure 3**.

T. tridentatus exhibits a broad distribution range, spanning multiple regions of East Asia. Its northern boundary starts along the coastal areas of Kyushu Island in Japan and then extends south along the coastline of southern China, encompassing the western part of Taiwan Island and Hainan Island. It further traverses the northern section of the Tonkin Gulf in Vietnam, reaching the Gulf of Thailand and continuing westward to the Andaman Sea. Its southern boundary stretches to the western coast of the Malay Peninsula and the island of Kalimantan. The core distribution areas of *T. tridentatus* are concentrated in the coastal regions of southern China and the Beibu Gulf, demonstrating the species' strong adaptability to these regions (see Figure 3A for details).

In contrast, the distribution range of *C. rotundicauda* is more southern-oriented. Its distribution starts on the western shore of the Taiwan Strait and extends north to cover the northern parts of the Tonkin Gulf and the coastal areas of the Gulf of Thailand. It further expands south, encompassing the coastal regions of the Malay Peninsula, Sumatra, and Kalimantan. The westernmost limit of its distribution reaches the northern coast of the Bay of Bengal. The core distribution areas of *C. rotundicauda* are located in the Gulf of Thailand, the coastal zones of the Malay Peninsula, and the coastal regions of Kalimantan, which are crucial for the survival of this species (**Figure 3B**).

Regarding *T. gigas*, its northern boundary in the South China Sea is roughly located off the coast of Nha Trang, Vietnam, and it can extend westward to the coastal waters along the

Western Ghats of the Indian Peninsula. Overall, the distribution range of *T. gigas* is quite extensive, starting from the southern part of the South China Sea and extending along the Gulf of Thailand, the Malay Peninsula, Kalimantan, Sumatra, and reaching as far as Java Island. It even bypasses the Bay of Bengal westward, reaching the coastal waters along the Western Ghats of the Indian Peninsula. The core distribution areas are located along the Gulf of Thailand and the Malay Peninsula (**Figure 3C**).

3.3 Key environmental factors influencing the potential habitats of *T. tridentatus*, *C. rotundicauda*, and *T. gigas*

For the three species of Asian horseshoe crabs, namely *T. tridentatus*, *C. rotundicauda*, and *T. gigas*, three of the most crucial environmental variables were selected for analysis as key environmental factors. Water depth, distance to land, and summer maximum chlorophyll-a concentration were identified as the pivotal factors influencing the potential habitat distribution of *T. tridentatus*. This species was found to inhabit waters with depths of up to -80 meters, within approximately 500 meters from the shore, and where the summer maximum chlorophyll-a concentration did not exceed 20 μg/L. The primary distribution was concentrated in waters within 0 to 200 meters from the shore and at depths of up to -40 meters (**Figure 4A-C**). For *C. rotundicauda*, water depth, distance to land, and minimum chlorophyll-a concentration emerged as the key factors shaping its potential habitat distribution. This species was distributed in waters with depths of up to -30 meters, within approximately 160 meters from the shore, and where the minimum chlorophyll-a concentration did not exceed 9 μg/L. The main distribution was focused in waters within 50 to 120 meters from the shore, at depths of up to -10 meters, and with minimum chlorophyll-a concentrations of 5 μg/L or less (**Figure 4D-F**). As for *T. gigas*, water depth, distance to land, and wind speed were determined to be the critical factors influencing its potential habitat distribution. This species was found in waters with depths of up to -70 meters, within approximately 500 meters from the shore, and where wind speeds did not exceed 5 m/s. The primary distribution was centered in waters within 50 to 200 meters from the shore, at depths of up to -40 meters, and with wind speeds of 1.5 m/s or less (**Figure 4G-I**).

3.4 Analysis of niche overlap and habitat similarity among three species of Asian

horseshoe crabs

The niche overlap coefficient, Schoener's D, between *T. tridentatus* and *C. rotundicauda* is 0.3203158, indicating a certain degree of overlap in ecological resources such as food and habitat, though not to a high degree of consistency (**Figure 5A**). This overlap may stem from their similar ecological needs and adaptation strategies, such as comparable positions in the food chain and similar habitat preferences. However, the habitat similarity p-value of 0.02398 suggests that, while there are commonalities in their habitats, significant differences also exist, which contribute to their coexistence and differentiation within the same ecosystem (**Figure 5B**). Additionally, the niche overlap coefficient between *T. tridentatus* and *T. gigas* is lower (Schoener's D = 0.2200121), and the habitat similarity p-value is even smaller ($p = 0.00999$), indicating more pronounced differences in their ecological needs and habitats (**Figure 5C-D**). These differences may arise from different adaptation strategies formed during evolution, such as differentiation in food choice and specialization of habitats, which help reduce interspecific competition and promote species coexistence. Conversely, the niche overlap coefficient between *C. rotundicauda* and *T. gigas* is higher (Schoener's D = 0.6221374), and the habitat similarity p-value is relatively low ($p = 0.01399$), indicating a higher similarity in ecological resources and habitats (**Figure 5E-F**). This high degree of niche overlap and habitat similarity may increase interspecific competition pressure between them, but it may also prompt them to develop more refined differentiation strategies in ecological adaptation and evolution, such as temporal niche partitioning and fine-grained division of food resources, to maintain population stability and ecological balance. *T. tridentatus*, *C. rotundicauda*, and *T. gigas* exhibit different characteristics in niche overlap and habitat similarity, which not only reflect their coexistence mechanisms within the same ecosystem but also provide important clues for deepening the understanding of interspecies interactions and evolutionary relationships.

3.5 Historical habitat distribution and refugia locations of Asian horseshoe crabs

The LGM had a decisive impact on the formation of current distribution patterns of Asian horseshoe crabs (*T. tridentatus*, *C. rotundicauda*, and *T. gigas*). **Figures 6A** to **6C** provide detailed illustrations of the lowly, moderately, and highly suitability habitats for these horseshoe crab species during the historical period, specifically the Last Glacial Maximum. Specifically,

for *T. tridentatus* (**Figure 6A**), during the LGM, its highly and moderately suitability habitats extended from the entrance of the Yellow River along the Chinese mainland coastline southwards, reaching the Beibu Gulf and northern Vietnam, and further expanding to the Mekong River estuary and the west coast of the Gulf of Thailand. During this period, the major refugia for *T. tridentatus* were concentrated in the Minjiang and Pearl River deltas, Leizhou Peninsula, and the northern coastal areas of the Beibu Gulf in China.

The historical distribution range of *C. rotundicauda* (**Figure 6B**) extended from the Indian Ocean coast to the Southeast Asian seas, west to the Indus River delta, passing through the northern Bay of Bengal, the Andaman Sea, crossing the Strait of Malacca, continuing eastward to the Gulf of Thailand, and ultimately reaching the waters surrounding the island of Kalimantan. The refugia for *C. rotundicauda* were scattered along both sides of the Strait of Malacca.

In contrast, the highly and moderately suitability habitats of *T. gigas* (**Figure 6C**) were more extensive, extending west near the Palk Strait near Sri Lanka, crossing the northern Bay of Bengal, the Andaman Sea, passing through the Strait of Malacca and the Gulf of Thailand, and ultimately reaching the waters surrounding the island of Kalimantan. Notably, large areas of potential suitable habitat were exhibited near the Java Sea. Its refugia were also located in the vicinity of the Strait of Malacca.

4. Discussion

4.1 Key environmental factors influencing the distribution of three species of Asian horseshoe crabs

The benthic marine environment, covering 70% of Earth's surface, holds fundamental importance within marine ecosystems (Tong et al., 2023). It provides food, shelter, and breeding grounds for numerous species (Buhl-Mortensen et al., 2010; Roberts et al., 2006) and offers a range of ecosystem services (Barbier et al., 2011). The Global Marine Environment Datasets (GMED) is currently one of the most authoritative databases available (Basher et al., 2018). In this study, 48 environmental factors—including 26 physical, 9 chemical, and 13 nutritional factors—extracted from the GMED database were used for modeling analysis after Variance Inflation Factor

(VIF) screening. Overall, water depth and distance to land are the most critical factors affecting the distribution of the three Asian horseshoe crabs, together shaping their distribution pattern. For different Asian horseshoe crab species, summer maximum chlorophyll-a, minimum chlorophyll-a concentration and wind speed affect their niche differences. Water depth is one of the significant factors influencing the distribution of marine habitats (Heres et al., 2024; Tong et al., 2023). For Asian horseshoe crabs, variations in water depth can potentially affect their survival and reproduction (Meilana et al., 2021). Different species of Asian horseshoe crabs may adapt to distinct water depth ranges. For instance, our research reveals that *C. rotundicauda* may prefer to inhabit shallower waters within -10 meters, whereas *T. tridentatus* and *T. gigas* may be more adapted to deeper environments within -40 meters. Changes in water depth can lead to alterations in the living space of horseshoe crabs, subsequently impacting their population sizes and distributions (Fairuz-Fozi et al., 2021). Furthermore, variations in water depth may also influence the food sources of horseshoe crabs (Liao et al., 2017; Wang et al., 2020). In deeper waters, horseshoe crabs may need to seek out more food sources to sustain their survival (Wang et al., 2020[illegible]he distance from the shore (distance to land) is also a crucial factor influencing the distribution of marine habitat, and variations in this distance can potentially impact the migration and reproduction patterns of marine organisms. The Asian horseshoe crab, for instance, may select different migration routes based on changes in the distance from the shore. Waters closer to the shore are more susceptible to human activities, such as pollution and fishing, which may compel horseshoe crabs to migrate to more distant waters. Furthermore, the distance from the shore can also affect the reproductive environment of horseshoe crabs. During their life cycles, *T. tridentatus*, *C. rotundicauda*, and *T. gigas* migrate in pairs to the vicinity of the high-tide zone in the intertidal zone to spawn (Koyama et al., 2022). The larvae of the horseshoe crab settle in nearshore muddy beaches, feeding on benthic organisms that are closely related to primary productivity, which is represented by chlorophyll-a. Consequently, chlorophyll-a content is actually a very important environmental factor that influences the distribution of horseshoe crabs, particularly the minimum chlorophyll-a concentration and the maximum

chlorophyll-a concentration during the summer breeding season. For the Asian horseshoe crab, specifically *T. gigas*, variations in wind speed primarily affect its activity range and survival condition. Strong winds may force the crabs to migrate to safer waters, whereas weaker winds may expand their activity range. Additionally, wind speed can potentially influence the reproduction success rate of the crabs. During the breeding season, strong winds may disrupt the reproductive environment of the horseshoe crabs, thereby reducing their reproduction success rate.

4.2 Niche overlap and resource utilization of three Asian horseshoe crabs

This study conducted an in-depth analysis of niche overlap and habitat similarity among three species of Asian horseshoe crabs: *T. tridentatus*, *C. rotundicauda*, and *T. gigas*. The results revealed complex relationships among these species in terms of ecological resource utilization and habitat selection, providing important insights into their coexistence mechanisms. The niche overlap coefficient between *T. tridentatus* and *C. rotundicauda* indicated that, despite a certain degree of overlap in resources such as food and habitat, there was not a high degree of consistency. This moderate overlap may reflect their similar ecological needs and adaptation strategies (Debnath et al., 2024), such as comparable positions in the food chain and similar habitat preferences (Tong et al., 2023). However, the p-value for habitat similarity showed that, despite commonalities, there were still significant differences in habitat selection between the two species. These differences may contribute to their coexistence in the same ecosystem and reduce direct competition through fine differentiation of niches. In contrast, the niche overlap coefficient between *T. tridentatus* and *T. gigas* was lower, and the p-value for habitat similarity was even smaller, indicating more significant differences in their ecological needs and habitats (Luo et al., 2023). These differences may stem from different adaptation strategies formed during evolution, such as differentiation in food choice and specialization of habitats. These strategies not only help reduce interspecific competition but may also promote species coexistence and diversity (Hemati et al., 2020). Notably, the niche overlap coefficient between *C. rotundicauda* and *T. gigas* was higher, while the p-value for habitat similarity was

relatively low. This high degree of niche overlap and habitat similarity may increase interspecific competition pressure between them. However, it may also prompt them to develop more refined differentiation strategies during ecological adaptation and evolution, such as temporal niche partitioning and fine-grained division of food resources. These strategies are crucial for maintaining population stability and ecological balance.

4.3 Distribution patterns of three species of Asian horseshoe crabs during the LGM

During the Quaternary Period, with the alternation between glacial and interglacial periods, flora and fauna were compelled to undergo large-scale migrations in order to adapt to climate changes, resulting in significant alterations in biodiversity distribution (Brown et al., 2020). Notably during the Last Glacial Maximum, when the climate reached its coldest phase and glaciers expanded to their greatest extents, glaciers in mountainous regions and high latitudes advanced to their maximum ranges, leading to substantial reduction or loss of habitats for numerous species (Barnosky, 2005). Consequently, some species migrated to new areas for survival, particularly marine organisms among them (Clark et al., 2009; Hughes, 2014). During the LGM, the potential habitats of *T. tridentatus* were widely distributed across a vast area from the entrance of the Yellow River to the Gulf of Thailand. This not only validates its robust environmental adaptability and migratory capacity but also suggests that significant changes in the marine environment during this period may have provided it with diverse habitat options. However, since the 1980s, records of *T. tridentatus* in China have been mainly confined to coastal waters of the East China Sea and South China Sea south of the Yangtze River Estuary (Sekiguchi and Sugita, 1980), indicating a contraction of its historical habitat range. The widespread distribution of *C. rotundicauda* along the Indian Ocean coast to Southeast Asian waters further supports its historical ability to survive and reproduce in various marine regions. Compared to historical times, the current habitats of *C. rotundicauda* have shifted slightly towards the southeast coast of China, indicating that the southeast coastal areas of China, located on the northern edge

of the South China Sea, represent the trailing edge of *C. rotundicauda*'s expansion, far from the central population. Habitats in this region may be more susceptible to environmental changes (Thorngren et al., 2019). During the LGM, *T. gigas* had extensive suitable habitats in the waters surrounding Indonesia and Malaysia near the equator, benefiting from sufficient heat at lower latitudes that shielded it from the abrupt temperature changes of the LGM (Clark et al., 2009).

The distribution characteristics of refugia for three Asian horseshoe crab species during the LGM provide vital clues for understanding their population dynamics. Refugia refer to areas that protect species from environmental changes and provide suitable conditions for survival (Brown et al., 2020; Hekinian and Walker, 1987). The refugia of *T. tridentatus* were mainly concentrated in coastal regions such as the Minjiang River and the Pearl River Delta. These areas likely possessed relatively stable environmental conditions and abundant food resources during the LGM, aiding the survival of its population in extreme environments. In contrast, the refugia of *C. rotundicauda* and *T. gigas* were scattered along both sides of the Strait of Malacca, potentially reflecting their more flexible survival strategies in response to environmental pressures. The Strait of Malacca features a tropical rainforest climate with high temperatures and abundant rainfall throughout the year, along with minimal wind (Goulding and Dayrat, 2023). The LGM was a cold period with a global temperature decline, resulting in a lower global sea level and potentially shallower waters in the Strait of Malacca compared to today (Alam et al., 2023; Goulding and Dayrat, 2023). This provided more habitats for the nearshore species, *C. rotundicauda* and *T. gigas*. This study offers beneficial insights for subsequent research. For instance, further exploration can be conducted on the migration pathways and population expansion mechanisms of the three Asian horseshoe crab species after the LGM, and how these processes have influenced their current distribution patterns. Additionally, paleoenvironmental data and molecular genetic techniques can be utilized to deeply investigate the response mechanisms and adaptation strategies of the three Asian horseshoe crab species to environmental changes, providing a more solid theoretical foundation for biodiversity conservation and ecosystem management.

4.4 Major threats to the current habitat of Asian horseshoe crabs

The habitats of Asian horseshoe crabs are facing increasingly severe pressures from human activities along the Indo-Pacific coast, which are complex and diverse, posing significant threats to their survival (Wang et al., 2020). Since the 1980s, to meet the commercial demand for bacterial endotoxin detection reagents (LAL/TAL), *L. polyphemus* and *T. tridentatus* have been captured extensively (Novitsky, 2015, 1994). Especially during the current coronavirus pandemic, with the surge in demand for vaccine testing and public health safeguards, the capture of these two species has further increased, resulting in sharp declines in their population sizes (Arnold, 2020). Among the four existing species of horseshoe crabs, *L. polyphemus* and *T. tridentatus* are classified as "Vulnerable (VU)" (Smith et al., 2017) and "Endangered (EN)" (Fang et al., 2023), respectively, on the IUCN Red List, while the other two, *T. gigas* and *C. rotundicauda*, are currently listed as "Data Deficient (DD)". However, reports indicate that the population of T. gigas is also declining (Nelson et al., 2016, 2015), and the widespread loss of mangrove habitats in Asia may have affected the presence of *C. rotundicauda* (Friess et al., 2012; Mishra et al., 2015).

Residential and commercial development in coastal areas is considered the primary threat to Asian horseshoe crab habitats. This development not only persists but may also have direct and irreversible impacts on coastal ecosystems (Crain et al., 2009; Defeo et al., 2009; Teichert et al., 2018). The expansion of human settlements, commercial and industrial developments, and agricultural and aquaculture activities along coastal lands have brought about large-scale landscape changes, further exacerbating the loss of horseshoe crab habitats. In Japan, concrete coastline protection structures along nesting sites have endangered suitable habitats for *T. tridentatus* (Botton et al., 2022). In Taiwan, China, the construction of armored shorelines, pollution, and invasive mangroves have negatively impacted horseshoe crab habitats, leading to local extinction of adult horseshoe crabs (Botton et al., 2022; Hsieh and Chen, 2015). Similarly, in many other coastal areas of Asia, including Hong Kong, China, and mainland China, shoreline reinforcement practices, such as the construction of bulkheads and seawalls, pose threats to Asian horseshoe crab habitats (Botton et al., 2022).

In addition to overcommercialization, coastal residential and commercial development,

Asian horseshoe crabs are confronted with threats such as "human intrusion and disturbance," "pollution," and "agriculture and aquaculture" (e.g., shrimp and finfish pond culture) (Wang et al., 2020). Furthermore, climate change, exploitation of biological resources, and alterations in natural systems are also recognized as significant factors influencing horseshoe crab habitats (Wang et al., 2020). Meanwhile, incidental bycatch and beach-combing activities pose additional threats. In the intertidal zones of Southeast Asia and southern China, numerous Asian horseshoe crabs are entangled in discarded fishing gear, which is often abandoned on shore (Fazrul et al., 2015). Beach-combing activities, especially during the peak spawning season of horseshoe crabs in summer, can cause severe disturbance to sediments, potentially imposing substantial impacts on horseshoe crabs and their benthic communities (Wang et al., 2020).

In summary, Asian horseshoe crabs are facing severe threats from multiple aspects, including overcommercialization, coastal residential and commercial development, human intrusion and disturbance, pollution, agriculture and aquaculture, climate change and ecosystem alterations, as well as incidental bycatch and beach-combing activities. These threats intertwine and collectively pose significant challenges to the survival of horseshoe crabs. Therefore, the formulation of effective conservation measures and strategies to address these threats is crucial for ensuring the survival and reproduction of horseshoe crab populations.

5. Conclusions

This study presents a comprehensive overview of the habitat characteristics, current potential habitat distributions with varying degrees of suitability, niche overlap and similarity between pairs, as well as the potential distribution patterns and refugia locations during the LGM of three Asian horseshoe crab species: *T. tridentatus*, *C. rotundicauda*, and *T. gigas*. The results indicate that water depth and distance to land are common key factors influencing the potential habitat distribution of these three Asian horseshoe crab species. However, different species are also affected by other specific environmental factors: the distribution of *T. tridentatus* is additionally constrained by the summer (May-Oct) maximum chlorophyll-a concentration, the potential habitat of *C. rotundicauda* is closely related to the minimum chlorophyll-a concentration, and habitat selection for *T. gigas* is significantly influenced by wind speed. In terms of niche overlap, *C. rotundicauda* and *T. gigas* exhibit the highest degree

of overlap (Schoener's D = 0.62), indicating high similarity in ecological resource utilization between them; in contrast, the niche overlap between *T. tridentatus* and *T. gigas* is the lowest (Schoener's D = 0.22), suggesting more pronounced niche differentiation. Reviewing the distribution patterns during the LGM, the refugia of *T. tridentatus* were mainly located at the Minjiang River and Pearl River estuaries, the surrounding seas of Leizhou Peninsula, and the northern part of the Beibu Gulf. The refugia of both *C. rotundicauda* and *T. gigas* were located near the Strait of Malacca, further emphasizing the crucial role of this region in horseshoe crab conservation. This study provides valuable insights into the conservation and management of these evolutionarily significant "living fossils," the horseshoe crabs.

Data availability statement

Data used in the study have been included in the article/Supplementary Material. For detailed information, please refer to the attached tables: *Tachypleus tridentatus*.XLS, *Tachypleus gigas*.XLS, and *Carcinoscorpius rotundicauda*.XLS.

Authorship contribution

Data curation: Jian Liao, Shui-Yuan Zhang, Zhong-Duo Wang

Investigation: Jian Liao, Shui-Yuan Zhang, Yu-Song Guo, Zhong-Duo Wang

Formal analysis: Jian Liao

Conceptualization: Jian Liao, Zhong-Duo Wang

Methodology: Jian Liao, Yu-Song Guo

Software: Jian Liao

Writing—original draft: Jian Liao

Writing—review and editing: Jian Liao, Chun-Hui Xiong, Gao-Cong Li, Jia-Yu Li, Yuan-Feng Yang, Shui-Yuan Zhang, Yi-Yang Li, Kai-Lin Zeng, Mei-Ling Hu, Zhong-Duo Wang

Supervision: Zhong-Duo Wang

Declaration of competing interest

The authors declare that they have no known competing financial interests or personal relationships that could have appeared to influence the work reported in this paper.

Funding

This study was funded by the Guangdong Provincial Field Observation and Research Station for Marine Ecosystem in Hanjiang River Estuary - Nanao Island Area Open Fund (Grant No. HNS202407), the National Key Research and Development Program of China (Grant No. 2024YFD2401803 & 2024YFD2401802), the Guangdong Provincial Ordinary University Youth Innovative Talent Project in 2024 (Grant No. 2024KQNCX134), the Guangdong Provincial Special Fund Project for Talent Development Strategy in 2024 (Grant No. 2024R3005), and the Guangdong Ocean University Scientific Research Startup Funding Project (Grant No. 060302022312).

References

Ahmad, R., Khuroo, A.A., Charles, B., Hamid, M., Rashid, I., Aravind, N.A., 2019. Global distribution modelling, invasion risk assessment and niche dynamics of Leucanthemum vulgare (Ox-eye Daisy) under climate change. Sci. Rep. 9, 11395. https://doi.org/10.1038/s41598-019-47859-1

Alam, L., Zolkaply, S.Z., Sumaila, U.R., Rusydy, I., Kutty, A.A., Bari, Md.A., Mokhtar, M., Pradhoshini, K.P., Krishnakumar, S., Musthafa, M.S., 2023. Risk of extinction, variability in fish species composition, and factors influencing fish biodiversity in the Malacca Strait. Environ. Sci. Pollut. Res. 31, 41355–41369. https://doi.org/10.1007/s11356-023-27101-2

Arnold, C., 2020. Horseshoe crab blood is key to making a COVID-19 vaccinedbut the ecosystem may suffer.

Barbier, E.B., Hacker, S.D., Kennedy, C., Koch, E.W., Stier, A.C., Silliman, B.R., 2011. The value of estuarine and coastal ecosystem services. Ecol. Monogr. 81, 169–193. https://doi.org/10.1890/10-1510.1

Barnosky, A.D., 2005. Effects of Quaternary Climatic Change on Speciation in Mammals. J. Mamm. Evol. 12, 247–264. https://doi.org/10.1007/s10914-005-4858-8

Basher, Z., Bowden, D.A., Costello, M.J., 2018. GMED: Global Marine Environment Datasets for environment visualisation and species distribution modelling. Earth Syst. Sci. Data Discuss. https://doi.org/10.5194/essd-2018-64

Botton, M.L., John, B.A., Carmichael, R.H., Mohamad, F., Bhadury, P., Zaldivar-Rae, J., Shin, P.K.S., Tanacredi, J.T., Cheung, S.G., 2022. Horseshoe Crabs: “Living Fossils” Imperiled in the Anthropocene, in: Imperiled: The Encyclopedia of Conservation. Elsevier, pp. 715–726. https://doi.org/10.1016/B978-0-12-821139-7.00103-3

Brown, S.C., Wigley, T.M.L., Otto-Bliesner, B.L., Rahbek, C., Fordham, D.A., 2020. Persistent Quaternary climate refugia are hospices for biodiversity in the Anthropocene. Nat. Clim. Chang. 10, 244–248. https://doi.org/10.1038/s41558-019-0682-7

Buhl-Mortensen, L., Vanreusel, A., Gooday, A.J., Levin, L.A., Priede, I.G., Buhl-Mortensen, P.,

Gheerardyn, H., King, N.J., Raes, M., 2010. Biological structures as a source of habitat heterogeneity and biodiversity on the deep ocean margins. Mar. Ecol. 31, 21–50. https://doi.org/10.1111/j.1439-0485.2010.00359.x

Carmichael, R.H., Botton, M.L., Shin, P.K.S., Cheung, S.G. (Eds.), 2015. Changing Global Perspectives on Horseshoe Crab Biology, Conservation and Management. Springer International Publishing, Cham. https://doi.org/10.1007/978-3-319-19542-1

Clark, P.U., Dyke, A.S., Shakun, J.D., Carlson, A.E., Clark, J., Wohlfarth, B., Mitrovica, J.X., Hostetler, S.W., McCabe, A.M., 2009. The Last Glacial Maximum. Science 325, 710–714. https://doi.org/10.1126/science.1172873

Crain, C.M., Halpern, B.S., Beck, M.W., Kappel, C.V., 2009. Understanding and Managing Human Threats to the Coastal Marine Environment. Ann. N. Y. Acad. Sci. 1162, 39–62. https://doi.org/10.1111/j.1749-6632.2009.04496.x

Debnath, S., Sarkar, U.K., Kumari, S., Karnatak, G., Puthiyottil, M., Das, B.K., Das, A., Ghosh, B.D., Roy, A., 2024. Exploring the vulnerability of the coastal wetlands of India to the changing climate and their adaptation strategies. Int. J. Biometeorol. https://doi.org/10.1007/s00484-024-02620-w

Defeo, O., McLachlan, A., Schoeman, D.S., Schlacher, T.A., Dugan, J., Jones, A., Lastra, M., Scapini, F., 2009. Threats to sandy beach ecosystems: A review. Estuar. Coast Shelf Sci. 81, 1–12. https://doi.org/10.1016/j.ecss.2008.09.022

Di Cola, V., Broennimann, O., Petitpierre, B., Breiner, F.T., D’Amen, M., Randin, C., Engler, R., Pottier, J., Pio, D., Dubuis, A., Pellissier, L., Mateo, R.G., Hordijk, W., Salamin, N., Guisan, A., 2017. ecospat: an R package to support spatial analyses and modeling of species niches and distributions. Ecography 40, 774–787. https://doi.org/10.1111/ecog.02671

Du, Z., Wu, J., Meng, X., Li, J., Huang, L., 2017. Predicting the Global Potential Distribution of Four Endangered Panax Species in Middle-and Low-Latitude Regions of China by the Geographic Information System for Global Medicinal Plants (GMPGIS). Molecules 22, 1630. https://doi.org/10.3390/molecules22101630

Elith, J., Phillips, S.J., Hastie, T., Dudík, M., Chee, Y.E., Yates, C.J., 2011. A statistical explanation of MaxEnt for ecologists: Statistical explanation of MaxEnt. Divers. Distrib. 17, 43–57. https://doi.org/10.1111/j.1472-4642.2010.00725.x

Fairuz-Fozi, N., Triest, L., Mat Zauki, N.A., Kaben, A.M., Nelson, B.R., Chatterji, A., Akhir, M.F., Satyanarayana, B., Dahdouh-Guebas, F., 2021. Mangrove horseshoe crab (*Carcinoscorpius rotundicauda* Latreille, 1802) populations reveal genetic break in Strait of Malacca, with connectivity along southern coasts of Peninsular Malaysia. Aquat. Conserv.-Mar. Freshw. Ecosyst. 31, 1559–1569. https://doi.org/10.1002/aqc.3552

Fang, J.K.-H., Tse, T.W., Maboloc, E.A., Leung, R.K.-L., Leung, M.M.-L., Wong, M.W.-T., Chui, A.P.-Y., Wang, Y., Hu, M., Kwan, K.Y., Cheung, S.G., 2023. Adverse impacts of high-density microplastics on juvenile growth and behaviour of the endangered tri-spine horseshoe crab Tachypleus tridentatus. Marine Pollution Bulletin 187, 114535. https://doi.org/10.1016/j.marpolbul.2022.114535

Fazrul, H., Hajisamae, S., Ikhwanuddin, Mhd., Pradit, S., 2015. Assessing Impact of Crab Gill Net Fishery to Bycatch Population in the Lower Gulf of Thailand. Turk. J. Fish. Aquat. Sci. 15. https://doi.org/10.4194/1303-2712-v15_3_21

Friess, D.A., Phelps, J., Leong, R.C., Lee, W.K., Wee, A.K.S., 2012. Mandai mangrove, Singapore:

lessons for the conservation of southeast asia’s mangroves. Raffles Bull. Zool. 25, 55–65.

Goulding, T.C., Dayrat, B., 2023. The Coral Triangle and Strait of Malacca are two distinct hotspots of mangrove biodiversity. Sci. Rep. 13, 15793. https://doi.org/10.1038/s41598-023-42057-6

Hekinian, R., Walker, D., 1987. Diversity and spatial zonation of volcanic rocks from the East Pacific Rise near 21° N. Contrib. Mineral. Petrol. 96, 265–280. https://doi.org/10.1007/BF00371248

Hemati, T., Pourebrahim, S., Monavari, M., Baghvand, A., 2020. Species-specific nature conservation prioritization (a combination of MaxEnt, Co$ting Nature and DINAMICA EGO modeling approaches). Ecol. Model. 429, 109093. https://doi.org/10.1016/j.ecolmodel.2020.109093

Heres, B., Abeels, H., Shea, C., Crowley-McIntyre, C.E., 2024. Environmental variables driving horseshoe crab spawning behavior in a microtidal lagoon in Florida. PLoS One 19, e0302433. https://doi.org/10.1371/journal.pone.0302433

Hsieh, H.-L., Chen, C.-P., 2015. Current status of *Tachypleus tridentatus* in Taiwan for Red List assessment. In: Carmichael, R., Botton, M., Shin, P., Cheung, S. (Eds.), Changing Global Perspectives on Horseshoe Crab Biology, Conservation and Management. Springer International, Switzerland.

Hughes, P.D., 2014. A stratigraphical basis for the Last Glacial Maximum (LGM). Quat. Int. 383, 174–185. https://doi.org/10.1016/j.quaint.2014.06.006

IUCN, 2024. The IUCN Red List of Threatened Species. https://www.iucnredlist.org

John, B.A., Nelson, B.R., Sheikh, H.I., Cheung, S.G., Wardiatno, Y., Dash, B.P., Tsuchiya, K., Iwasaki, Y., Pati, S., 2018. A review on fisheries and conservation status of Asian horseshoe crabs. Biodivers. Conserv. 27, 3573–3598. https://doi.org/10.1007/s10531-018-1633-8

Koyama, A., Kawahara, Y., Kurita, Y., Hirata, T., Hayashi, O., Takahashi, S., Kubozono, H., Onikura, N., Minagawa, T., 2022. Assessment of spawning events of the tri-spine horseshoe crab, *Tachypleus tridentatus*, using environmental DNA in the Sone tidal flat, Japan. Plankton Benthos Res. 17, 127–136. https://doi.org/10.3800/pbr.17.127

Le Pape, O., Bonhommeau, S., Nieblas, A.-E., Fromentin, J.-M., 2017. Overfishing causes frequent fish population collapses but rare extinctions. Proc. Natl. Acad. Sci. U.S.A. 114. https://doi.org/10.1073/pnas.1706893114

Liao, J., Wang, H., Xiao, S., Guan, Z., Zhang, H., Dumont, H.J., Han, B.-P., 2022. Modeling and prediction of the species’ range of *Neurobasis chinensis* (Linnaeus, 1758) under climate change. Biology 11, 868. https://doi.org/10.3390/biology11060868

Liao, J., Wu, Z., Wang, H., Xiao, S., Mo, P., Cui, X., 2023. Projected Effects of Climate Change on Species Range of Pantala flavescens, a Wandering Glider Dragonfly. Biology 12, 226. https://doi.org/10.3390/biology12020226

Liao, Y., Hsieh, H.-L., Xu, S., Zhong, Q., Lei, J., Liang, M., Fang, H., Xu, L., Lin, W., Xiao, X., Chen, C.-P., Cheung, S.G., Kwan, B.K.Y., 2017. Wisdom of Crowds reveals decline of Asian horseshoe crabs in Beibu Gulf, China. Oryx 53, 222–229. https://doi.org/10.1017/S003060531700117X

Ling, L.P., Adibah, A.B., Tan, S.G., Christianus, A., Faridah, Q.Z., 2013. Isolation by the 5′ anchored PCR technique and characterization of eighteen microsatellite loci in horseshoe crab (Tachypleus gigas). J. Genet. 92, 101–104. https://doi.org/10.1007/s12041-011-0115-5

Luo, W., Han, S., Yu, T., Wang, P., Ma, Y., Wan, M., Liu, J., Li, Z., Tao, J., 2023. Assessing the suitability and dynamics of three medicinal Sambucus species in China under current and future climate scenarios. Front. Plant Sci. 14, 1194444. https://doi.org/10.3389/fpls.2023.1194444

Meilana, L., Hakim, A.A., Fang, Q., 2021. Nursery habitat of three species of juvenile Asian horseshoe crabs in Teritip Beach, East Kalimantan, Indonesia: Characterization and implication. Glob. Ecol. Conserv. 26, e01453. https://doi.org/10.1016/j.gecco.2021.e01453

Mishra, J.K., Mishra, A., Yasmin, 2015. Status and threat perceptions of the Indian horseshoe crabs along the northeast coast of bay of Bengal, India. In: Carmichael, R.H., Botton, M.L., Shin, P.K.S., Cheung, S.G. (Eds.), Changing Global Perspectives on Horseshoe Crab Biology, Conservation and Management. Springer, Cham.

Nelson, B.R., Satyanarayana, B., Moh, J.H.Z., Ikhwanuddin, M., Chatterji, A., Shaharom, F., 2016. The final spawning ground of *Tachypleus gigas* (Müller, 1785) on the east Peninsular Malaysia is at risk: a call for action. PeerJ 4, e2232. https://doi.org/10.7717/peerj.2232

Nelson, B.R., Satyanarayana, B., Zhong, J.M.H., Shaharom, F., Sukumaran, M., Chatterji, A., 2015. Episodic human activities and seasonal impacts on the Tachypleus gigas (MUller, 1785) population at Tanjung Selangor in Peninsular Malaysia. Estuar. Coast Shelf Sci. 164, 313–323. http://dx.doi.org/10.1016/j.ecss.2015.08.003

Nong, W., 2021. Horseshoe crab genomes reveal the evolution of genes and microRNAs after three rounds of whole genome duplication. Commun. Biol. 4, 1–11. https://doi.org/10.1038/s42003-020-01637-2

Novitsky, T.J., 2015. Biomedical implications for managing the *Limulus polyphemus* harvest along the northeast coast of the United States. In: Carmichael, R. H., Botton, M.L., Shin, P.K.S., Cheung, S.G. (Eds.), Changing Global Perspectives on Horseshoe Crab Biology. Springer, Gland, Switzerland.

Novitsky, T.J., 1994. Limulus amebocyte lysate (LAL) detection of endotoxin in human blood. J. Endotoxin Res. 1, 253–263. https://doi.org/10.1177/096805199400100407

Phillips, S.J., Anderson, R.P., Schapire, R.E., 2006. Maximum entropy modeling of species geographic distributions. Ecol. Model. 190, 231–259. https://doi.org/10.1016/j.ecolmodel.2005.03.026

Pshegusov, R., Chadaeva, V., 2023. Modelling the nesting-habitat of threatened vulture species in the caucasus: An ecosystem approach to formalising environmental factors in species distribution models. Avian Res. 14, 100131. https://doi.org/10.1016/j.avrs.2023.100131

Roberts, J.M., Wheeler, A.J., Freiwald, A., 2006. Reefs of the Deep: The Biology and Geology of Cold-Water Coral Ecosystems. Science 312, 543–547. https://doi.org/10.1126/science.1119861

Rudkin, D.M., Young, G.A., 2009. Horseshoe crabs – an ancient ancestry revealed, in: Biology and Conservation of Horseshoe Crabs. Springer New York, US, pp. 25–44.

Sekiguchi, K., Sugita, H., 1980. Systematics and hybridization in the four living species of horseshoe crabs. Evolution 34, 712–718. https://doi.org/10.1111/j.1558-5646.1980.tb04010.x

Sheikh, H.I., John, B.A., J.A. Ichwan, S., B.Y., K., 2021. Effect of prolonged captivity on the hemolymph profile of Tachypleus gigas using the various anticoagulant formulations. Aquacult. Rep. 20, 100760. https://doi.org/10.1016/j.aqrep.2021.100760

Smith, D.R., Brockmann, H.J., Beekey, M.A., King, T.L., Millard, M.J., Zaldívar-Rae, J., 2017. Conservation status of the American horseshoe crab, (Limulus polyphemus): a regional assessment. Rev Fish Biol Fisheries 27, 135–175. https://doi.org/10.1007/s11160-016-9461-y

Stirling, D.A., Boulcott, P., Scott, B.E., Wright, P.J., 2016. Using verified species distribution models to inform the conservation of a rare marine species. Divers. Distrib. 22, 808–822. https://doi.org/10.1111/ddi.12447

Tanacredi, J.T., Botton, M.L., Smith, D. (Eds.), 2009. Biology and Conservation of Horseshoe Crabs. Springer US, Boston, MA. https://doi.org/10.1007/978-0-387-89959-6

Teichert, N., Carassou, L., Sahraoui, Y., Lobry, J., Lepage, M., 2018. Influence of intertidal seascape on the functional structure of fish assemblages: Implications for habitat conservation in estuarine ecosystems. Aquat. Conserv. Mar. Freshw. Ecosyst. 28, 798–809. https://doi.org/10.1002/aqc.2914

Thorngren, L., Bergström, P., Dunér Holthuis, T., Lindegarth, M., 2019. Assessment of the population of *Ostrea edulis* in Sweden: A marginal population of significance? Ecol. Evol. 9, 13877–13888. https://doi.org/10.1002/ece3.5824

Tong, R., Yesson, C., Yu, J., Luo, Y., Zhang, L., 2023. Key factors for species distribution modeling in benthic marine environments. Front. Mar. Sci. 10, 1222382. https://doi.org/10.3389/fmars.2023.1222382

Wang, C., Huang, S., Wang, X., Xu, P., Huang, X., Liao, Y., Xie, X., Kwan, K., 2019. Conserving the understudied invertebrates: a call for a systematic monitoring protocol for Asian horseshoe crabs in nursery habitats. Endang. Species. Res. 40, 369–373. https://doi.org/10.3354/esr01003

Wang, C.-C., Kwan, K.Y., Shin, P.K.S., Cheung, S.G., Itaya, S., Iwasaki, Y., Cai, L., Mohamad, F., Fozi, N.F., Mat Zauki, N.A., Raman, N.J.A., Chatterji, A., Tripathy, B., Sajan, S., Min, W.W., Tan, L.J.X., Supadminingsih, F.N., Wardiatno, Y., Hsieh, H.-L., 2020. Future of Asian horseshoe crab conservation under explicit baseline gaps: A global perspective. Global Ecology and Conservation 24, e01373. https://doi.org/10.1016/j.gecco.2020.e01373

Xiong, K., Liu, X., Xie, Z., Waiho, K., Fang, J.K.H., Wang, Y., Hu, M., 2023. The use of physiological and transcriptional analyses to examine molting regulatory mechanisms in juvenile horseshoe crab Tachypleus tridentatus. Aquaculture 572, 739518. https://doi.org/10.1016/j.aquaculture.2023.739518

Yi, Y., Cheng, X., Yang, Z.-F., Zhang, S.-H., 2016. Maxent modeling for predicting the potential distribution of endangered medicinal plant (H. riparia Lour) in Yunnan, China. Ecol. Eng. 92, 260–269. https://doi.org/10.1016/j.ecoleng.2016.04.010

Zhang, K., Yao, L., Meng, J., Tao, J., 2018. Maxent modeling for predicting the potential geographical distribution of two peony species under climate change. Sci. Total Environ. 634, 1326–1334. https://doi.org/10.1016/j.scitotenv.2018.04.112

Zhao, R., Wang, S., Chen, S., 2024. Predicting the potential habitat suitability of Saussurea species in China under future climate scenarios using the optimized Maximum Entropy (MaxEnt) model. J. Clean Prod. 474, 143552. https://doi.org/10.1016/j.jclepro.2024.143552

Zhu J., Wu Z., Feng B., Deng S., Zhen W., Liao Y., Xie X., Yue Kwan K., 2020. Global conservation of *Tachypleus tridentatus*: Present status and recommendations. Biodiversity Science 28, 621–629. https://doi.org/10.17520/biods.2019401

Figure

Figure 1. Distribution Maps of *T. tridentatus*, *C. rotundicauda*, and *T. gigas*

Figure 2. Importance analysis of environmental variables for the habitats of *T. tridentatus* (A)*,* *C. rotundicauda* (B)*, and T. gigas* (C) based on Jackknife Test

Figure 3. Distribution of potential habitats with different suitability levels for *T. tridentatus* (A), *C. rotundicauda* (B), and *T. gigas* (C).

Figure 4. Scatter plots of key environmental factors versus suitability values for the potential habitats of *T. tridentatus*, *C. rotundicauda*, and *T. gigas*. (A) Depth vs. suitability value for *T. tridentatus*, (B) Distance to land vs. suitability value for *T. tridentatus*, (C) Maximum chlorophyll-a in summer vs. suitability value for *T. tridentatus*, (D) Depth vs. suitability value for *C. rotundicauda*, (E) Distance to land vs. suitability value for *C. rotundicauda*, (F) Minimum chlorophyll-a vs. suitability value for *C. rotundicauda*, (G) Depth vs. suitability value for *T. gigas*, (H) Distance to land vs. suitability value for *T. gigas*, (I) Wind speed vs. suitability value for *T. gigas*.

Figure 5. Niche Overlap and Similarity *T. tridentatus*, *C. rotundicauda*, and *T. gigas*. (A) Niche Overlap between *T. tridentatus* and *C. rotundicauda*, (B) Niche Similarity between *T. tridentatus* and *C. rotundicauda*, (C) Niche Overlap between *T. tridentatus* and *T. gigas*, (D) Niche Similarity between *T. tridentatus* and *T. gigas*, (E) Niche Overlap between *C. rotundicauda* and *T. gigas*, (F) Niche Similarity between *C. rotundicauda* and *T. gigas*.

Figure 6. Potential habitat distribution and refuge locations with different degrees of suitability for *T. tridentatus* (A), *C. rotundicauda* (B), and *T. gigas* (C) during the Last Glacial Maximum.

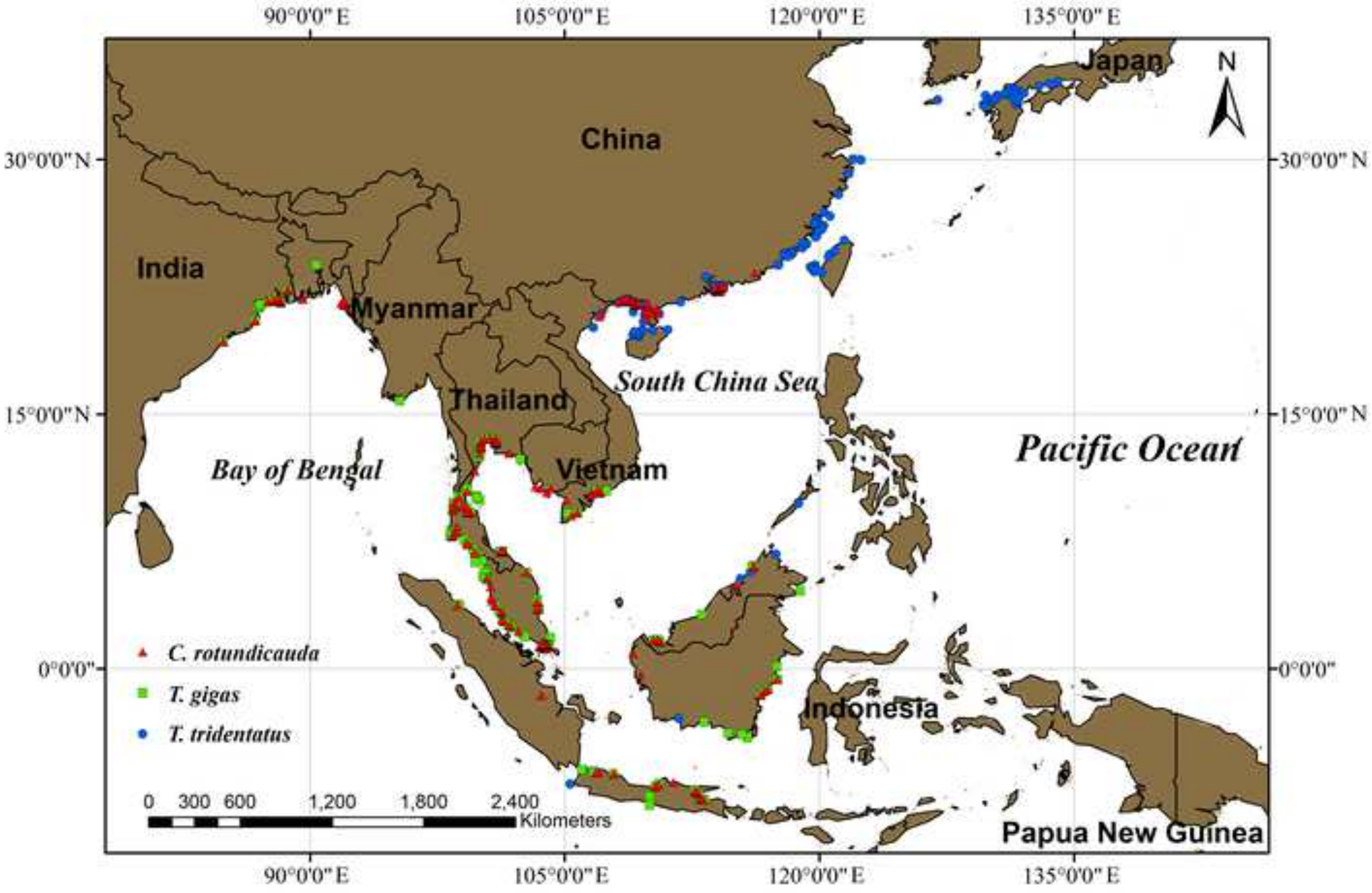
90°0'0" E
105°0'0" E
120°0'0" E
135°0'0" E
30°0'0" N
15°0'0" N
0°0'0"
Japan
N
China
India
Myanmar
South China Sea
Thailand
Pacific Ocean
Bay of Bengal
Vietnam
C. rotundicauda
T. gigas
T. tridentatus
Indonesia
0 300 600 1,200 1,800 2,400
Kilometers
Papua New Guinea

Importance analysis of environmental variables for the habitats of T. tridentatus (A), C. rotundicauda (B), and T. gigas (C) based on

Click here to access/download;Figure;Figure 2.tif

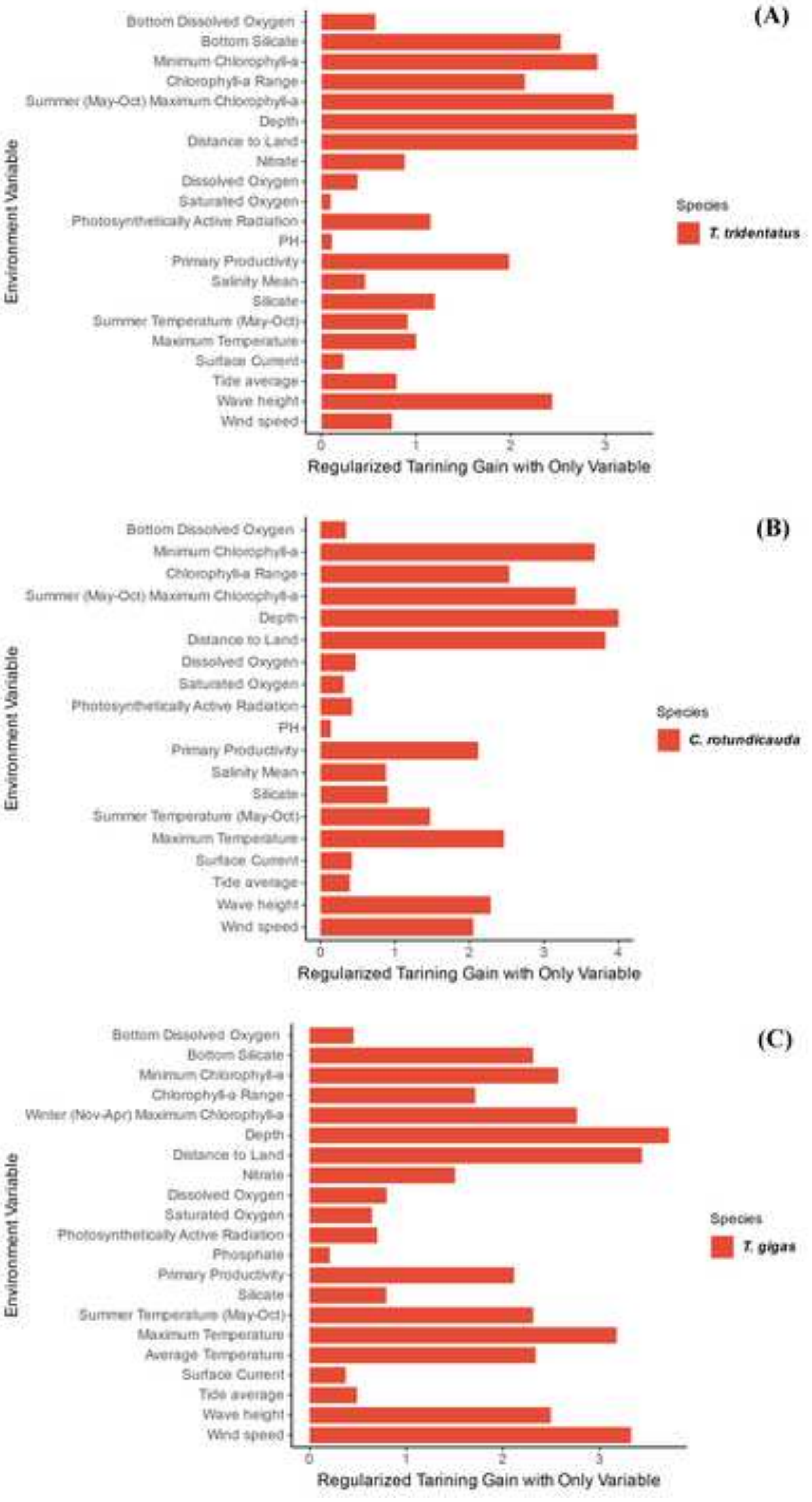

Distribution of potential habitats with different suitability levels for T. tridentatus (A), C. rotundicauda (B), and T. gigas (C).

Click here to access/download;Figure;Figure 3A.tif

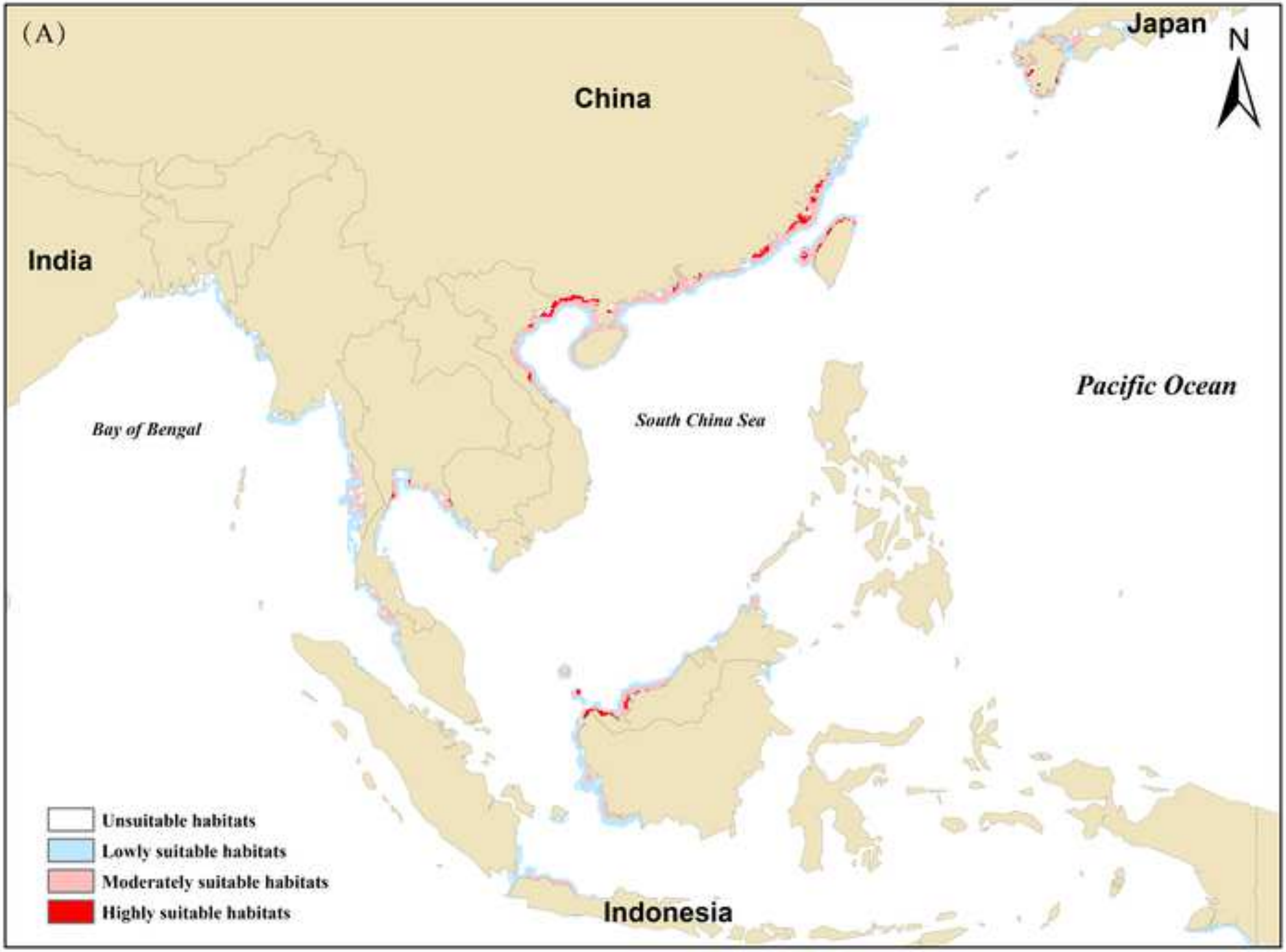

Distribution of potential habitats with different suitability levels for T. tridentatus (A), C. rotundicauda (B), and T. gigas (C)

Click here to access/download;Figure;Figure 3B.tif

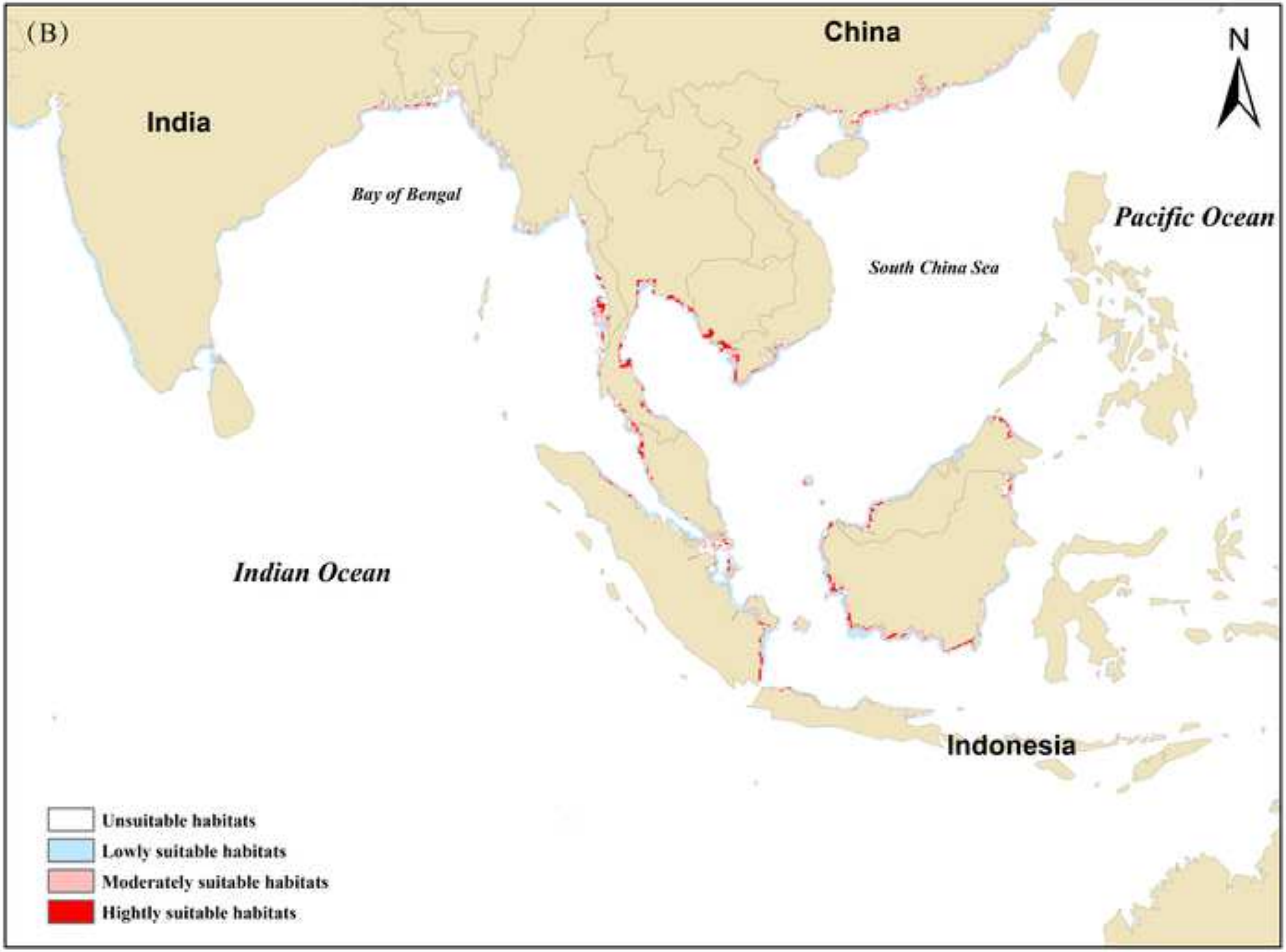

Distribution of potential habitats with different suitability levels for T. tridentatus (A), C. rotundicauda (B), and T. gigas (C)

Click here to access/download;Figure;Figure 3C.tif

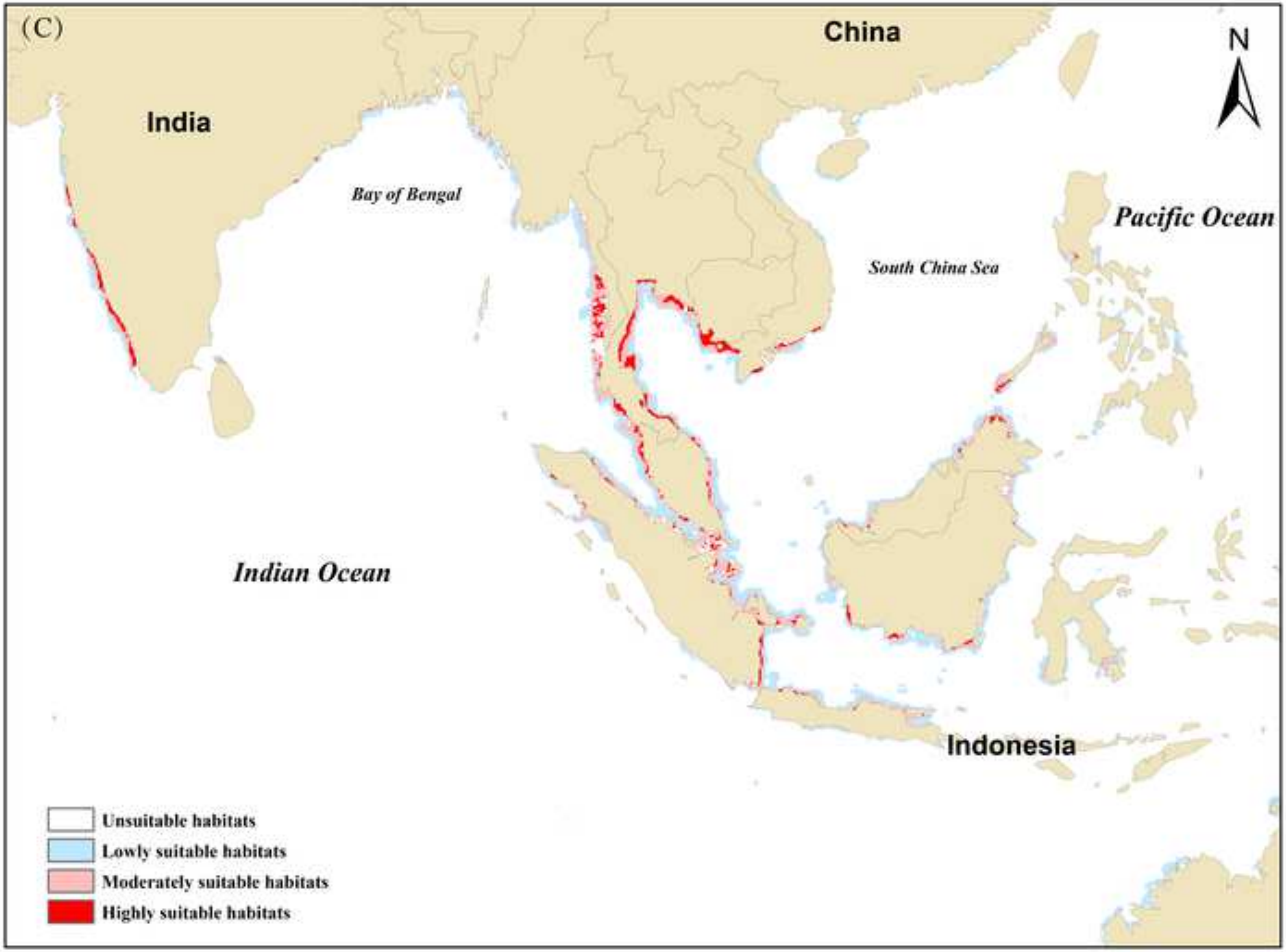

Scatter plots of key environmental factors versus suitability values for the potential habitats of T. tridentatus, C. rotundicauda, and T.

Click here to access/download;Figure;Figure 4.tif

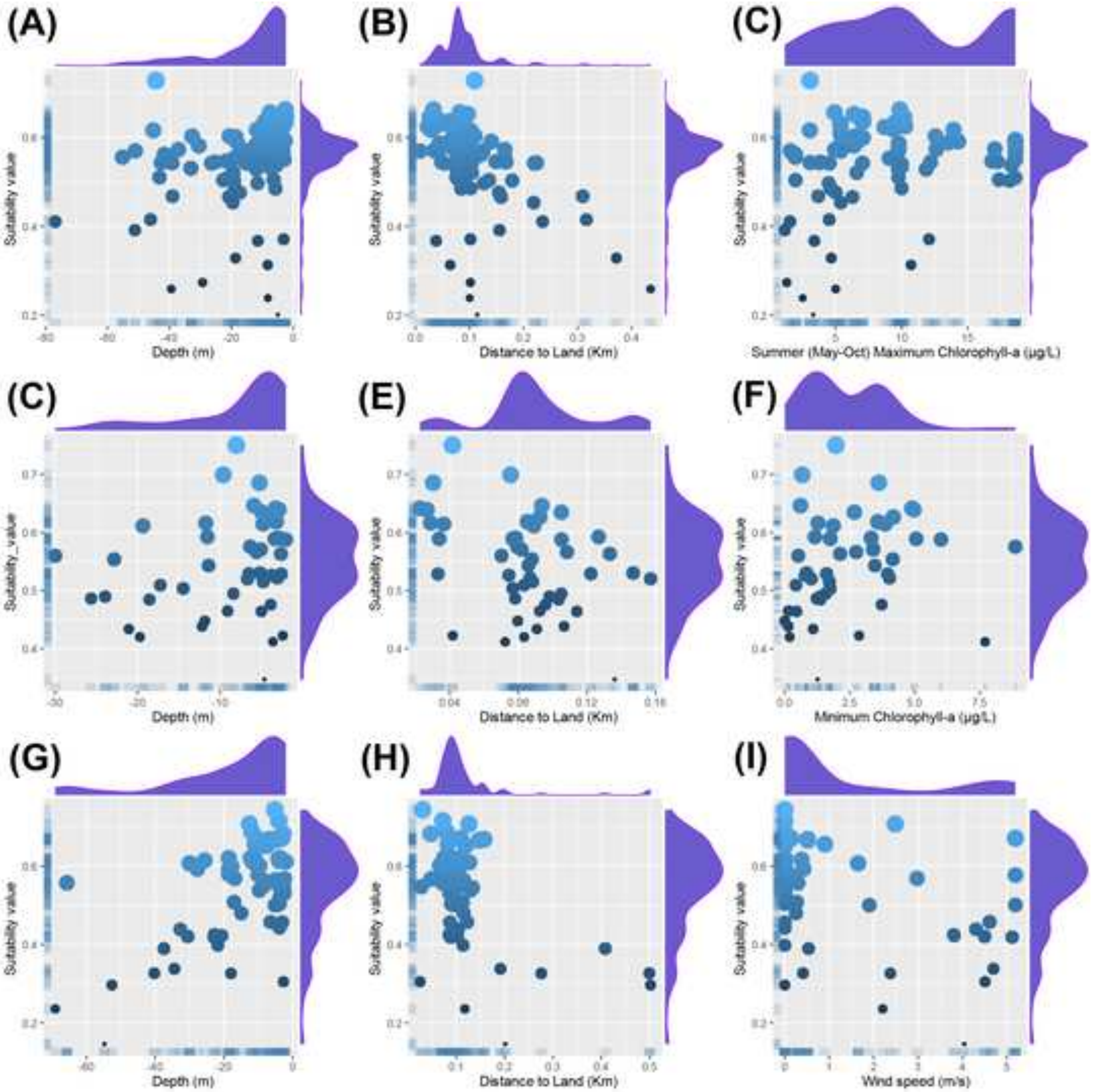

Niche Overlap and Similarity T. tridentatus, C. rotundicauda, and T. gigas. (A) Niche Overlap between T. tridentatus and C.

Click here to access/download;Figure;Figure 5.tif

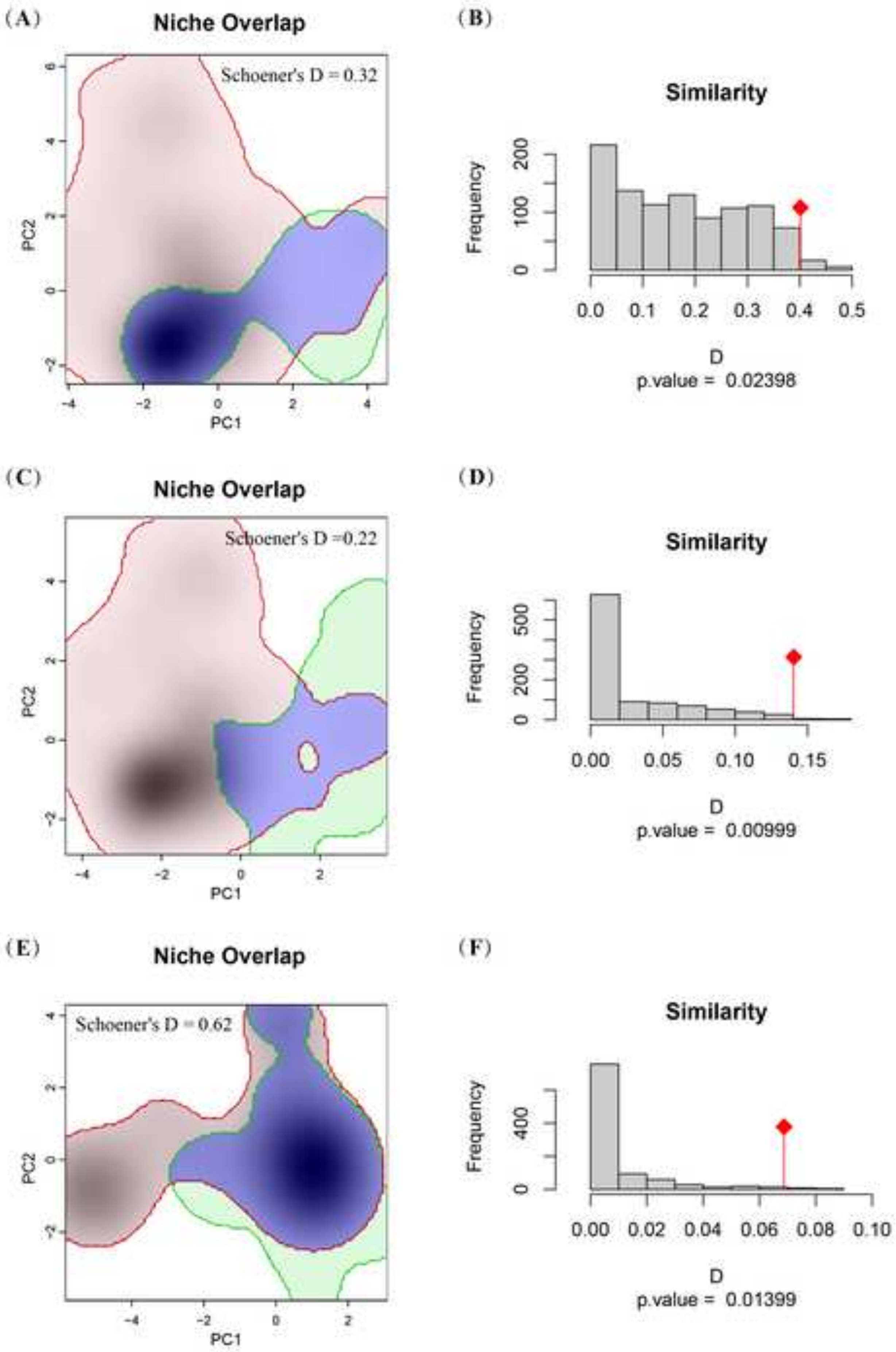

Potential habitat distribution and refuge locations with different degrees of suitability for T. tridentatus (A), C. rotundicauda (B), and T. gigas (C) during the Last Glacial

Click here to access/download;Figure;Figure 6A.tif

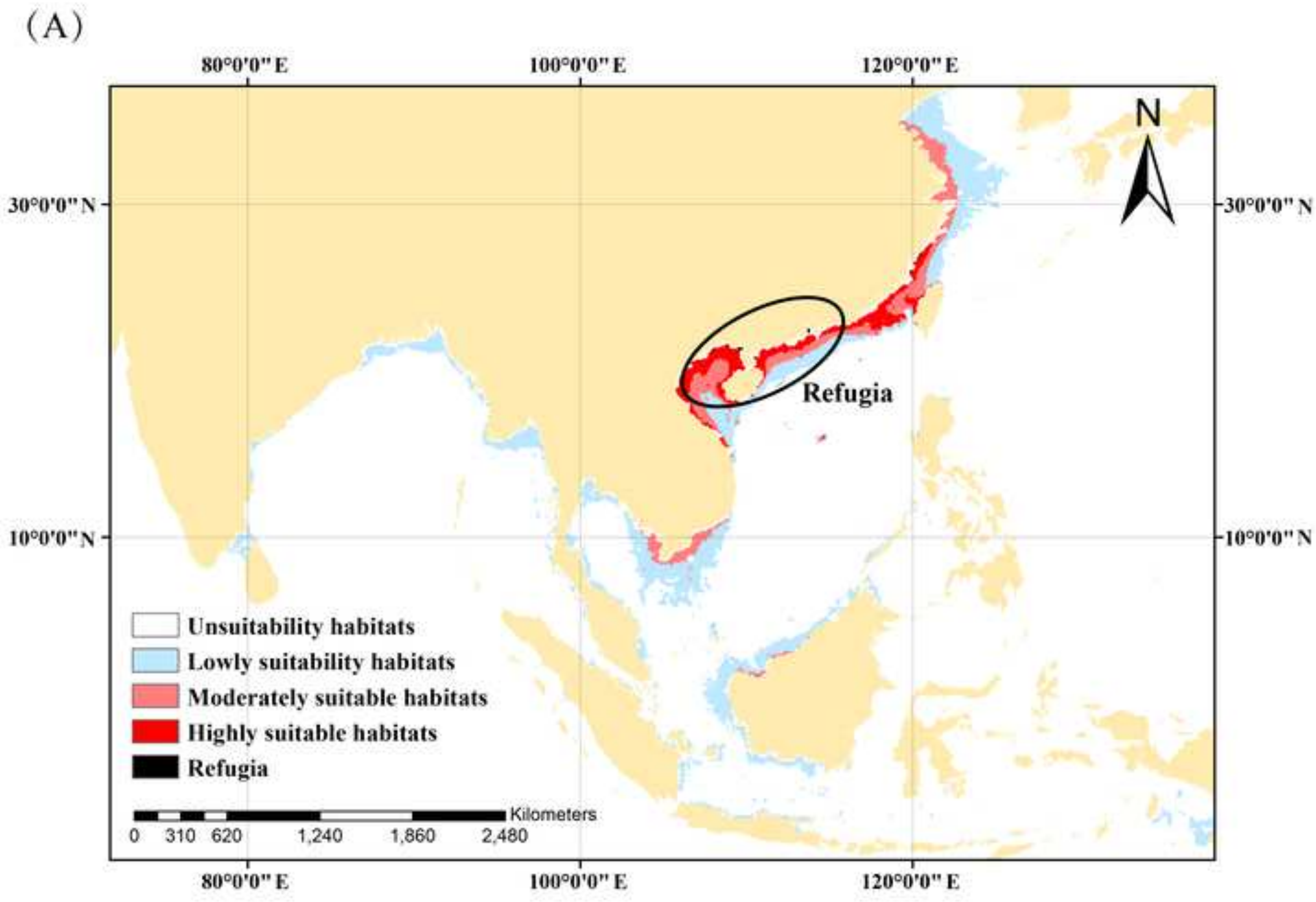

Potential habitat distribution and refuge locations with different degrees of suitability for T. tridentatus (A), C. rotundicauda (B), and T. gigas (C) during the Last Glacial

Click here to access/download;Figure;Figure 6B.tif

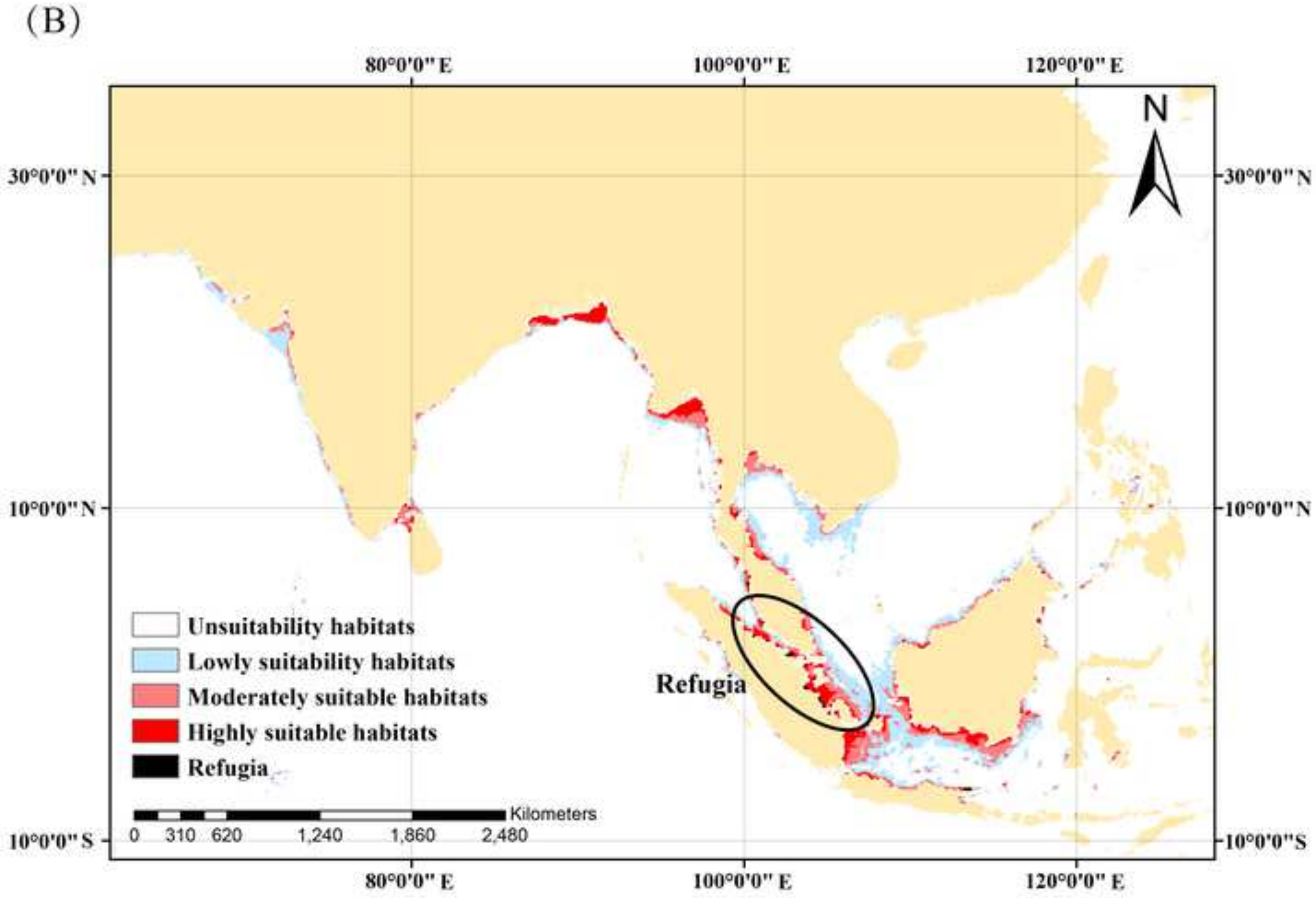

Potential habitat distribution and refuge locations with different degrees of suitability for T. tridentatus (A), C. rotundicauda (B), and T. gigas (C) during the Last Glacial

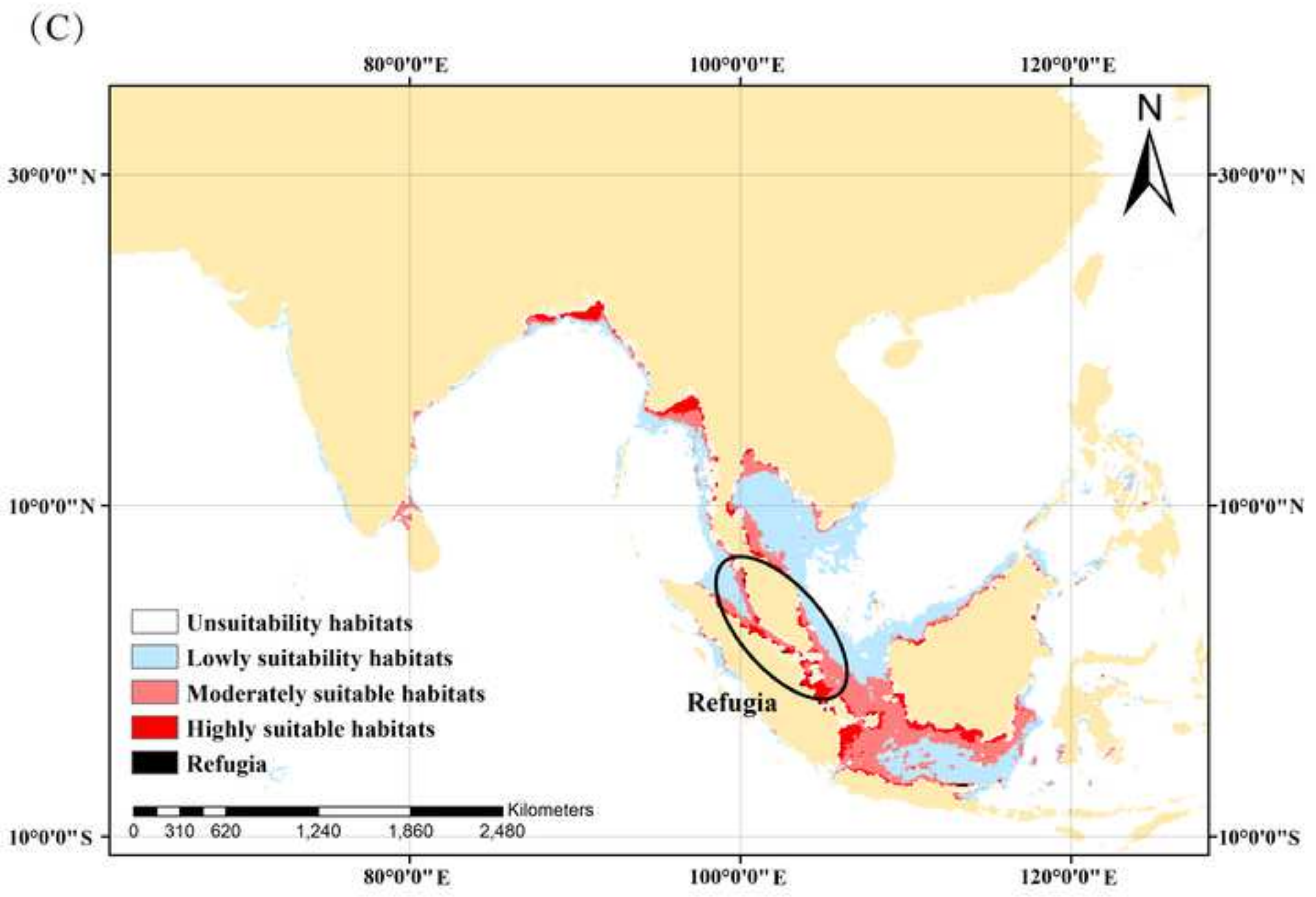

Click here to access/download
Supporting Information - Compressed/ZIP File Archive
Supporting Information.zip
