# Supplementary material for: Modeling habitat distribution and niche overlap of Asian horseshoe crabs: Implications for conservation
Source: PLoS One. 2025 May 28;20(5):e0324471. doi: 10.1371/journal.pone.0324471 (PMC12118861; doi:10.1371/journal.pone.0324471)
Supplement: Attachment — Submitted filename: Review (15Mar2025) - Horse crabs.pdf [file pone.0324471.s005.pdf]

Manuscript PONE-D-25-08646

Dear *Dr. Phuping Sucharitakul*,

Thank you for the invitation to review the manuscript *"Exploring habitat characteristics, distribution, and niche relationships of Asian horseshoe crabs: preserving living fossils and biodiversity" (PONE-D-25-08646).* After a detailed reading, I provide my assessment of the study and suggestions for its improvement below.

General Recommendations

The study presents a robust analysis of the distribution and habitat characteristics of Asian horseshoe crabs (*Tachypleus tridentatus, Tachypleus gigas,* and *Carcinoscorpius rotundicauda*), employing predictive modeling to infer both historical and contemporary patterns. The use of the MaxEnt model and niche overlap metrics are well-established and appropriate methodological approaches for the study's objectives.

Despite its scientific relevance and potential impact on species conservation, the manuscript has some weaknesses, including: 1) a lack of deeper justifications for certain methodological choices; 2) some model-based inferences that require stronger support from the literature; and 3) minor textual adjustments to improve clarity and argumentation.

Thus, I recommend that the manuscript undergo revision before acceptance for publication, incorporating the suggested adjustments outlined below.

Manuscript Evaluation and Scoring

Criterion	Score (1 to 5)	Comment
**Originality**	4	The study addresses a relevant and underexplored topic but could provide a more in-depth discussion of the implications of the findings for species conservation.
**Methodology**	4	The use of the MaxEnt model is appropriate, but some environmental variable selections lack justification. Additionally, the robustness of the species occurrence data could be more thoroughly detailed.
**Clarity and Organization**	3.5	Some sections of the manuscript are dense and could be better structured to improve readability. It is suggested to revise portions of the Introduction and Discussion to enhance their flow.
**Interpretation of Results**	4	The results are interesting and well-presented, but some inferences, particularly regarding niche overlap, require stronger support from additional references.
**Scientific Impact and Relevance**	4.5	The study contributes to conservation biology and can inform public policies, but the connection between findings and practical conservation applications could be more strongly emphasized.

Specific Comments

Justification for Environmental Variables: The selection of environmental variables for the MaxEnt model appears coherent, but the manuscript should provide a stronger rationale for how each variable impacts species ecology. Additionally, a brief explanation of variable correlation (multicollinearity) would be useful, as some variables may have redundant influences on the model.

Niche Overlap Analyses: The study uses metrics such as Schoener's index to estimate niche overlap. However, the results could be better contextualized with previous studies that have used similar approaches. The manuscript mentions habitat overlap between *T. gigas* and *C. rotundicauda*, but it lacks a clearer explanation of the ecological factors driving this interaction.

Text Organization: The Introduction is well-detailed but could be more concise. Some information about horseshoe crab biology is repetitive and could be consolidated into a single paragraph. In the Discussion, it is recommended to structure paragraphs more clearly to distinguish obtained results from inferences and comparisons with other studies.

Conservation and Applicability: The manuscript raises important conservation concerns but does not sufficiently explore how the findings could be applied in developing management strategies. It would be valuable to include practical recommendations based on the results.

Grammatical and Stylistic Revisions

It is suggested to review the writing to improve clarity and flow, particularly in lengthy sections of the Introduction and Discussion. Additionally, some technical terms could be explained more accessibly for readers from other conservation biology fields.

Additional Suggestions

- If possible, include an analysis of uncertainty in the predictive models, discussing the limitations of the approach used.
- Expand the literature review to incorporate more recent studies on horseshoe crab conservation and anthropogenic impacts on their habitats.
- Adjust figure captions to make them more self-explanatory.

Conclusion

Manuscript PONE-D-25-08646 is a relevant and well-conducted study on the ecology and conservation of Asian horseshoe crabs. However, some methodological and structural aspects need refinement to strengthen its conclusions. I recommend revisions before acceptance, as outlined above.

Sincerely,

Prof. Dr. Marcelo Antônio Amaro Pinheiro
UNESP IB/CLP – Brazil
marcelo.pinheiro@unesp.br

CONTRIBUTION TO AUTHORS

SUGGESTION FOR TITLE

In my view, the title "**Exploring habitat characteristics, distribution, and niche relationships of Asian horseshoe crabs: preserving living fossils and biodiversity"** is relatively long and could be more focused on the main topics addressed. Therefore, I suggest the title **"Modeling habitat distribution and niche overlap of Asian horseshoe crabs: glacial refugia and conservation priorities"** which incorporates niche modeling and overlap, as well as the significance of glacial refugia for conservation.

ABSTRACT

The abstract is well-structured but could be improved, particularly by clarifying the implications of the results and strengthening the connection between the findings and conservation. Thus, I suggest structuring the abstract as follows

"Asian horseshoe crabs are ancient organisms essential for the balance of marine ecosystems. However, detailed information on their ecology and the environmental factors influencing their distribution remains limited. In this study, we analyzed habitat characteristics, potential distribution, niche overlap, and refugia during the Last Glacial Maximum (LGM) for three species: *Tachypleus tridentatus*, *Carcinoscorpius rotundicauda*, and *Tachypleus gigas*. Predictive modeling using MaxEnt and niche analysis revealed that water depth and proximity to land are key factors determining species distribution, with species-specific environmental influences: *T. tridentatus* is affected by maximum summer chlorophyll-a, *C. rotundicauda* by minimum chlorophyll-a, and *T. gigas* by wind velocity. Niche overlap was highest between *C. rotundicauda* and *T. gigas*, while *T. tridentatus* showed lower overlap with the other species. During the LGM, refugia for *T. tridentatus* were concentrated in the Minjiang and Pearl River estuaries, while *C. rotundicauda* and *T. gigas* found shelter in the Malacca Strait region. The results highlight priority conservation areas, providing insights for management and protection strategies amid current environmental threats."

INTRODUCTION

The introduction has a solid and well-founded structure, contextualizing the importance of Asian horseshoe crabs, their threats, and the need for predictive modeling in conservation. However, it can be improved in terms of fluency, reduction of redundancies, and making the text more direct and impactful. The main suggested changes are as follows:

Page 2, Lines 45-54: The idea that horseshoe crabs are "living fossils" and that their morphology has remained unchanged for millions of years is repeated. This information is already mentioned earlier in the introduction. Therefore, I suggest a more concise mention to avoid redundancy: "Horseshoe crabs, whose morphology has remained virtually unchanged for over 200 million years, play a fundamental role in marine ecosystems and biodiversity maintenance."

Page 3, Lines 60-68: The relevance of horseshoe crabs in marine ecology is reiterated similarly to previous statements. This should either be removed or reformulated to highlight new aspects of their ecological function, such as their interactions with food chains or environmental vulnerability.

Page 3, Lines 77-88: The transition between threats and predictive modeling is abrupt. The text shifts from habitat degradation directly to MaxEnt modeling without a clear connection. I suggest adding a transition sentence explaining how modeling can aid in mitigating environmental impacts and conserving the species: "Given habitat degradation and the need to identify priority conservation areas, species distribution models become essential tools. MaxEnt, widely used in ecological studies, enables highly accurate predictions of species distribution."

Page 4, Lines 105-113: The study objectives are presented, but no mention of a testable hypothesis is made. I suggest adding: "We hypothesize that water depth, land distance, and specific environmental factors are the primary determinants of the distribution of the three Asian horseshoe crab species. We use the MaxEnt model to predict potential habitats and analyze niche overlap, aiming to provide insights for species conservation."

MATERIALS AND METHODS

This section is well-structured, adequately detailing data collection, predictive modeling, and statistical analysis. The inclusion of Table 1, which presents important environmental variables and their corresponding Variance Inflation Factor (VIF) values for the three species, is excellent. However, I suggest a few improvements:

Page 5, Lines 120-130: The text does not mention how potential errors in occurrence records, such as inaccurate or outdated coordinates, were handled. I suggest adding: "In addition to removing duplicates and anomalous points, records with inaccurate coordinates or located in terrestrial environments were manually verified and corrected whenever possible."

Page 8, Lines 153-155: The arbitrary criterion for splitting training (75%) and testing (25%) data is not justified. It would be important to insert this justification, such as: "The 75/25 (training/testing) split was chosen as it is widely used in ecological modeling studies, ensuring a balance between adequate model training and robust validation."

Page 8, Lines 155-157: The text states that the MaxEnt model was run until convergence but does not mention whether adjustments for regularization, maximum iterations, pseudo-absence numbers, or other key parameters were made. Therefore, I suggest including: "MaxEnt parameters were adjusted to minimize overfitting, using a regularization value of X, a maximum iteration number of Y, and 10,000 randomly generated pseudo-absences within the study area."

Page 8, Lines 172-176: It would be important to explain why the Schoener index was used to measure niche overlap. I suggest adding: "The Schoener index (D) was chosen as it is widely used in ecological niche studies, providing a robust measure of niche overlap by considering both presence differences and habitat suitability intensity."

RESULTS

The Results section provides detailed and consistent information on the habitat modeling of the three Xiphosura species, with excellent organization and statistical robustness. However, some improvements can enhance clarity, avoid redundancies, and strengthen data interpretation. Below are some suggestions for improvement:

Page 9, Lines 184-189: AUC values are repeatedly presented for each species, which could be synthesized into a table for easier reading. Therefore, I suggest including the following text and table: "AUC values were above 0.99 for all species (*T. tridentatus*: 0.9963/0.9958, *C. rotundicauda*: 0.9980/0.9972, *T. gigas*: 0.9974/0.9972), indicating high model reliability (Table ??)."

Page 9, Lines 194-196: The text lists key variables but does not explain their ecological importance for each species. I suggest including: "Water depth may be related to the availability of spawning substrate, while chlorophyll-a reflects primary productivity, influencing food availability. Wind velocity may affect larval dispersal and sediment transport."

Page 12, Lines 273-284: The D coefficient is presented, but its implication for species coexistence (biological significance of niche overlap) could be improved. Suggested revision: "The low overlap between *T. tridentatus* and *T. gigas* suggests ecological segregation, possibly due to distinct habitat and dietary preferences. In contrast, the high overlap between *C. rotundicauda* and *T. gigas* may indicate stronger competition for resources, requiring additional niche differentiation mechanisms."

Page 13, Lines 298-303: The study demonstrates that species had distinct refugia during the LGM but does not discuss how this may influence conservation strategies. Suggested addition: "The identification of refugia suggests that these areas may harbor genetically resilient populations, making them conservation priorities."

FIGURES

The figures effectively visualize spatial distribution patterns and ecological niches of the species and are well-structured. Below are some improvements to enhance clarity and accessibility of the data:

Figure 1: Recommend labeling key geographic regions mentioned in the text, such as the Malacca Strait and the Gulf of Thailand.

Figure 2: Each species' variables could be represented by distinct colors (lighter shades), with the three most important variables for each species highlighted in more contrasting (darker) colors. Another suggestion is to place the species' names directly on each graph instead of using a legend (red square followed by species name).

Figure 3 (A, B, and C): Legends could be more intuitive by using a more perceptible color gradient (e.g., yellow for low suitability, orange for moderate suitability, and red for high suitability). Additionally, indicating key ecological areas directly on the maps would facilitate more direct access to information.

Figure 4: Clarity could be improved by standardizing the axes for the same relationships, allowing direct comparison between the three species, with points colored as in Figure 2. This would enable comparisons of the three species for each column, placing titles above the left column—Depth

(m)—and the middle column—Land Distance (km)—which would compose Figure 4 (letters A, B, D, E, G, and H). Chlorophyll-a (Maximum or Summer ...) would form another figure, while Wind Velocity (m/s) would be isolated as another figure. This restructuring could enhance readability. Alternatively, all graphs could remain in Figure 4 but grouped within frames labeled with the variable name. Two vertical columns with three graphs each (left and center) and two graphs (chlorophyll-a) with another for wind velocity would be displayed. Maintaining the same axis values for comparable variables is crucial, with distinct color points representing each species (as suggested for Figure 2).

Figure 5: Could include a graphical representation of niche distribution in environmental space for better interpretation. Additionally, a bar graph illustrating absolute niche overlap values would be beneficial.

Figure 6 (A, B, and C): The obtained results do not allow for arrows indicating possible post-LGM species dispersal routes. Another relevant aspect would be overlaying current distributions to facilitate past and present comparisons.

DISCUSSION

The Discussion section is well-structured, addressing the study's main findings and relating them to previous research. However, some aspects could be improved to make the text more concise, enhance its flow, and strengthen the connection between the results and species conservation.

Page 14, Line 353 and Page 15, Line 358-364: The text mentions the importance of depth and chlorophyll-a but does not detail how these factors directly influence horseshoe crab behavior and survival. Suggested addition: "Depth influences not only the availability of suitable spawning substrate but also access to food sources and protection from predators. Similarly, chlorophyll-a reflects primary productivity, impacting the abundance of organisms forming the horseshoe crab food base."

Page 15, Lines 367-376: The study presents niche overlap coefficients among species but could better explore the conservation and management implications of these results. Suggested addition: "The high overlap between *C. rotundicauda* and *T. gigas* may indicate greater competitive pressure, requiring additional strategies for habitat differentiation or temporal reproductive shifts to reduce ecological conflicts. This information can inform management policies to prevent degradation of critical shared habitats."

Page 17, Lines 424-447: Identifying refugia during the LGM is an important aspect of the study, but the text does not explore how this information can be useful for current conservation. I strongly recommend expanding this section. One possible addition: "The identification of historical refugia suggests areas that potentially harbor genetically resilient populations. Protecting these regions may be crucial for ensuring species population viability in future climate change scenarios."

Page 18, Lines 450-463: The discussion assertively lists threats to Asian Xiphosura species, such as habitat destruction and overfishing, but does not suggest concrete measures to mitigate these impacts. It would be expected for the authors to mention this. Suggested addition: "The implementation of marine protected areas and monitoring programs is recommended to assess population responses to environmental impacts. Furthermore, regulating the fishing and biomedical

harvesting of horseshoe crabs should be strengthened to ensure a balance between conservation and the sustainable use of this biological resource."

CONCLUSIONS

The conclusions could be substantially improved by a revision to avoid repetitions (e.g., repeating environmental factors such as chlorophyll-a and depth as influential factors) and to make the text more direct. Additionally, concrete conservation policy recommendations could be included (e.g., proposing the implementation of marine protected zones in areas identified as critical habitats and historical refuges, along with strengthening regulations on the harvest of these species for biomedical purposes, aiming for a balance between conservation and sustainable use). Moreover, the connection between historical distribution and future climate change could be emphasized (e.g., future analyses to assess the effects of global warming and ocean acidification on the distribution of horseshoe crabs, potentially shifting their habitats to new areas). Finally, the importance of MaxEnt modeling as a predictive tool for conservation should be highlighted (e.g., MaxEnt modeling identified habitat distribution patterns and provided key insights for conservation using predictive data, representing an advancement in the ecological understanding of these species).
