# Supplementary material for: Modeling habitat distribution and niche overlap of Asian horseshoe crabs: Implications for conservation
Source: PLoS One. 2025 May 28;20(5):e0324471. doi: 10.1371/journal.pone.0324471 (PMC12118861; doi:10.1371/journal.pone.0324471)
Supplement: Attachment — Submitted filename: Final Review - Marcelo Pinheiro.pdf [file pone.0324471.s007.pdf]

Final Review – Second Round of Revision

Manuscript: PONE-D-25-08646R1 – Modeling habitat distribution and niche overlap of Asian horseshoe crabs: Implications for Conservation
Reviewer: Marcelo A. A. Pinheiro
Date: April 11, 2025

1) General Assessment

After reviewing the revised manuscript and comparing it with the comments provided in the initial review, I conclude that the authors have fully addressed 80% of my suggestions and partially addressed an additional 13%, responding clearly and appropriately to all points raised. Only 7% of the recommendations were not incorporated; however, the authors' explanations for doing so were satisfactory.

The revised title is excellent, and a clear hypothesis has been appropriately included. The manuscript now provides justifications for using adult data and includes proper statistical treatment of environmental variables through VIF analysis. Significant improvements have been made to the abstract, discussion, and conclusion, which are now more concise and directly focused on the study's implications. I particularly appreciated the addition of references to support the role of wind and depth in benthic species distribution and the expanded ecological discussion on niche overlap among the studied species.

Regarding Figures 4 and 5, the authors tested my suggestions but ultimately did not adopt them due to limited visual effectiveness and graphic overlap issues, which the authors duly explained. The section addressing glacial refugia (LGM) and its conservation relevance was entirely removed. While initially suggested for expansion, I concur with the decision to omit this content, as it has improved the focus of the manuscript in alignment with the perspective of the other reviewers.

2) Final Considerations

Overall, the authors have demonstrated a genuine effort to incorporate the reviewers' feedback, resulting in substantial improvements in the structure, writing quality, ecological reasoning, and overall scientific robustness of the manuscript. The remaining limitations are minor and do not compromise the relevance or scientific merit of the study. I commend the authors for their collegial tone in the responses and for producing a high-quality contribution that is likely to be well-received and frequently cited in the field.

Recommendation: Accept.
