# Supplementary material for: Modeling habitat distribution and niche overlap of Asian horseshoe crabs: Implications for conservation
Source: PLoS One. 2025 May 28;20(5):e0324471. doi: 10.1371/journal.pone.0324471 (PMC12118861; doi:10.1371/journal.pone.0324471)
Supplement: Attachment — Submitted filename: Response to Reviewers.docx [file pone.0324471.s008.docx]

Journal Requirements:

When submitting your revision, we need you to address these additional requirements.

1. Please ensure that your manuscript meets PLOS ONE's style requirements, including those for file naming. The PLOS ONE style templates can be found at

<https://journals.plos.org/plosone/s/file?id=wjVg/PLOSOne_formatting_sample_main_body.pdf> and

[https://journals.plos.org/plosone/s/file?id=ba62/PLOSOne_formatting_sample_title_authors_affiliations.pdf](https://track.editorialmanager.com/CL0/https:%2F%2Fjournals.plos.org%2Fplosone%2Fs%2Ffile%3Fid=ba62%2FPLOSOne_formatting_sample_title_authors_affiliations.pdf/1/010f0195d03a03b4-933948eb-35a6-43d9-b8c6-c6e63a37fe8f-000000/NkMhJ9V2RYAHnEp3DP3zRbZy2IkYLdeiNOjh12c4d8w=204)

**Response：**Thank you for your reminder. We have made format revisions in accordance with your journal's requirements, including heading sizes, figure and table formats and specifications, reference formatting, and acknowledgments, among others.

2**.** Thank you for stating the following financial disclosure:
“This study was funded by the Guangdong Provincial Field Observation and Research Station for Marine Ecosystem in Hanjiang River Estuary - Nanao Island Area Open Fund (Grant No. HNS202407), the National Key Research and Development Program of China (Grant No. 2024YFD2401803 & 2024YFD2401802), the Guangdong Provincial Ordinary University Youth Innovative Talent Project in 2024 (Grant No. 2024KQNCX134), the Guangdong Provincial Special Fund Project for Talent Development Strategy in 2024 (Grant No. 2024R3005), and the Guangdong Ocean University Scientific Research Startup Funding Project (Grant No. 060302022312).”

Please state what role the funders took in the study. If the funders had no role, please state: "The funders had no role in study design, data collection and analysis, decision to publish, or preparation of the manuscript."
If this statement is not correct you must amend it as needed.
Please include this amended Role of Funder statement in your cover letter; we will change the online submission form on your behalf.

**Response：**Thank you for your reminder. The original manuscript already includes financial disclosure, and we have added "Funding acquisition: Jian Liao" to the author contributions in the revised version.

3. Please include captions for your Supporting Information files at the end of your manuscript, and update any in-text citations to match accordingly. Please see our Supporting Information guidelines for more information: <http://journals.plos.org/plosone/s/supporting-information>.

**Response：**Thank you for your suggestion. We have added a title to the supporting information file at the end of the manuscript and updated the in-text citations accordingly, including adding "(S1 to S3 File)" to the Environmental variables section in the Materials and Methods.

**Reviewer #1:** The manuscript presents a valuable analysis of the distribution and habitat characteristics of three Asian horseshoe crab species using MaxEnt modeling and niche analysis. The use of MaxEnt modeling is appropriate for species distribution predictions, and the study follows standard procedures for model validation (e.g., AUC assessment, VIF analysis to remove collinearity).

The incorporation of paleo-environmental modeling to assess Last Glacial Maximum (LGM) refugia is a valuable approach for understanding historical species distributions.

The study has strong potential for contributing to conservation efforts by identifying key environmental factors affecting species distribution. However, there are major issues with how the findings are framed, interpreted, and connected to the broader context of conservation and biodiversity management. The manuscript would benefit from a stronger positioning within the state of the art, improved discussion of methodological assumptions, and clearer conservation implications. The Introduction does not sufficiently compare the study’s approach and findings with prior research on horseshoe crab distribution. The manuscript should clearly specify:

--What aspects of habitat suitability and niche relationships have already been studied for these species.

What specific gaps this study addresses (e.g., finer-scale niche overlap analysis, previously overlooked environmental factors).

-How the findings advance current understanding.

**Response：**Thank you for your attentive comments. We have added a short paragraph at the end of the third section in the introduction, elaborating on the research of marine organisms, especially horseshoe crab habitat suitability and ecological niches, and pointing out that the environmental variables they used were climatic variables, which have a weak correlation with horseshoe crabs, a marine benthic organism. We have comprehensively considered marine environmental factors with greater impact, including physical, chemical, and nutritional variables, offering predictable advantages (Line 122-132). The specific added content is as follows:

For example, the MaxEnt model has been used to simulate scad fish (Decanters spp.) in the northern South China Sea [26], and joint species distribution models have been employed to predict the distribution of seafloor taxa and identify vulnerable marine ecosystems in New Zealand waters [27]. Regarding research on horseshoe crab habitat suitability and suitability relationships, Chinese scholars have conducted local studies in the Beibu Gulf region [28]. However, the environmental factors studied were limited to climatic factors, which have a weak correlation with horseshoe crabs, a marine benthic organism [28]. Our study, starting from the marine environment, comprehensively considers physical, chemical, and nutritional factors, which are more in line with the habitat conditions of horseshoe crabs, a marine benthic organism, and offers predictable advantages.

The Discussion and Conclusion sections claim that the study supports conservation strategies but fail to articulate:

How the identified key environmental factors should inform conservation efforts.

Specific recommendations for habitat protection or management actions.

Potential policy implications (e.g., marine protected areas, restoration initiatives).

**Response：**Thank you for your valuable comments. In response to your feedback, we have made targeted additions to the Discussion and Conclusion sections to address the points raised (). Specifically, we have proposed the following conservation and regulatory measures:

1. Establishment of Marine Protected Areas (MPAs): We recommend the creation of MPAs in critical habitats, with a particular focus on areas with high habitat overlap, to protect shared ecological resources. This measure aims to safeguard these vital ecosystems and the species that depend on them.

2. Strengthening Regulations on Harvesting: We emphasize the need for stricter regulations on the harvest of horseshoe crabs for biomedical purposes. This is crucial to balance conservation efforts with sustainable use, ensuring that these species are not overexploited for short-term gains.

3. Proactive Strategies for Climate Change Impacts: Recognizing the potential impacts of global warming and ocean acidification on horseshoe crab distributions, we propose proactive strategies to assess these effects. This includes monitoring potential habitat shifts to new areas due to changing environmental conditions, such as alterations in chlorophyll-a concentrations and wind speed. By doing so, we can inform adaptive conservation measures and ensure that protection efforts remain effective in the face of changing environmental conditions.

These additions provide a comprehensive framework for conservation and management, addressing the need for specific recommendations and policy implications. We believe these measures will contribute to the long-term survival and sustainability of horseshoe crab populations and their ecosystems.

The manuscript does not clarify whether occurrence data correspond to adults, juveniles, or larvae, which is critical for interpreting distribution patterns. Horseshoe crabs exhibit ontogenetic habitat shifts, with larvae often requiring different environmental conditions than adults.

**Response：**Thank you for pointing out this important issue. We used distribution data from adults, which are more accessible. Adult horseshoe crabs exhibit more stable habitat characteristics and more pronounced activity patterns, making their distribution data more systematically acquirable through field surveys, satellite tracking, and other means. In existing databases and literature records, the spatial and temporal continuity of adult distribution records is also more complete. We have added supplementary explanations in the corresponding sections, such as in the "Species occurrence sites" subsection within the Materials and Methods section (Line 149).

Specific comments are found in the attached pdf and must be addressed individually.

The title does not clearly reflect the study contents; there is no attempt to addressing results with species and biodiversity preservation. instead focus on the aims novelty in relation to state of the art

**Response：**Thank you for your constructive feedback. We have carefully considered your comments in conjunction with those from another reviewer, and have revised the manuscript accordingly. In response to your specific concern regarding the title, we have modified it to better reflect the study contents and their implications for conservation. The new title, "Modeling habitat distribution and niche overlap of Asian horseshoe crabs: Implications for Conservation," explicitly links our research on habitat distribution modeling and niche overlap analysis to their conservation implications (Line 3-5). This revision aims to emphasize our study's contribution to understanding the ecological requirements of Asian horseshoe crabs and providing valuable insights for their conservation and management. We appreciate your attention to detail and your efforts in helping us improve the manuscript.

These claims are not supported by the manuscript, particularly the discussion lacks any contextualisation of results and conservation measures. REformulate the take home message in terms of contribution to gaps in state of the art

**Response：**Thank you for your valuable feedback. We have enhanced both the discussion and conclusion sections with the following content:

Discussion:

To effectively address the pressing conservation challenges outlined above and mitigate the threats to Asian horseshoe crabs, it is imperative to implement a combination of protective measures and sustainable management strategies. The implementation of marine protected areas and monitoring programs is recommended to assess population responses to environmental impacts. Furthermore, regulating the fishing and biomedical harvesting of horseshoe crabs should be strengthened to ensure a balance between conservation and the sustainable use of this biological resource.

Conclusion:

To enhance protection, we recommend establishing marine protected areas (MPAs) in identified critical habitats, particularly focusing on regions where habitat overlap is high, to safeguard shared ecological resources. Additionally, strengthening regulations on the harvest of these species for biomedical purposes is essential to balance conservation with sustainable use. Proactive strategies are needed to assess the impacts of global warming and ocean acidification on horseshoe crab distributions. This includes monitoring potential habitat shifts to new areas due to changing environmental conditions, such as alterations in chlorophyll-a concentrations and wind speed, to inform adaptive conservation measures.

these are repeated from the title, make them more useful to target readers by choosing other words, perhaps focusing on different info

**Response：**Thank you for your valuable feedback. Following the suggestion of another reviewer, we have updated the title to: "Modeling habitat distribution and niche overlap of Asian horseshoe crabs: Implications for Conservation". Additionally, to avoid repetition between the title and the keywords, we have replaced the keywords "*Tachypleus tridentatus*, *Tachypleus gigas*, *Carcinoscorpius rotundicauda*, Potential habitats, Niche overlap, Biodiversity" with "Horseshoe crabs, Species distribution models, Key environmental factors, Niche, Biodiversity" (Line 57-58). These revisions aim to enhance clarity and focus on the core aspects of our study. We appreciate your time and consideration.

The introduction does not clearly position the study contribution in relation to the state of the art for the tested aspects. a great focus should be given to that here.

**Response：**Thank you for your valuable feedback. In response to your comments, we have revisited the introduction section to more clearly articulate the contribution of our study in relation to the current state of the art. Specifically, in Lines 122-136, we have provided a detailed discussion on the advantages of MaxEnt technology, its application in horseshoe crab research, and the existing limitations. Furthermore, we would like to highlight the main contribution of our research. We have overcome the limitations of using conventional low-relevance climate data by incorporating marine environmental parameters, including physical, chemical, and nutritional variables, for the first time in horseshoe crab distribution prediction. This approach provides essential data for their conservation. We believe these revisions have strengthened the manuscript and made our contribution more evident. Thank you again for your helpful suggestions.

These expected contributions do not match the discussion and conclusion contents. delete of change to match.

**Response：**Thank you for your valuable feedback. We have carefully revised the text to ensure that the expected contributions align with the discussion and conclusion contents. We have deleted the sections that were not strongly relevant and made necessary modifications to better highlight the contributions and focus of our study.

The revised text now reads as follows:

As horseshoe crabs are typical intertidal benthic organisms, we hypothesize that water depth, land distance, and specific environmental factors are the primary determinants of the distribution of the three Asian horseshoe crab species. We use the MaxEnt model to predict potential habitats and analyze niche overlap, aiming to provide insights for horseshoe crab conservation. This study also offers methods and ideas for the conservation research of other endangered species, contributing to the advancement of global biodiversity conservation efforts (Line 133-145).

We believe these revisions have strengthened the manuscript and made our contributions more evident. Thank you again for your helpful suggestions.

it is very important to clarify here if the crabs are adults, or different life stages as that should influence results interpretation.

**Response：**Thank you for your insightful feedback. We have taken care to specify in the manuscript that the horseshoe crabs examined in our study are adults (Line 149). This clarification is crucial, as it directly impacts the interpretation of our findings and ensures their accuracy. To prevent any ambiguity, we have explicitly highlighted this detail in the pertinent sections of the text, thereby providing a transparent understanding of the life stage of the horseshoe crabs under investigation. Your constructive suggestions have been invaluable in refining our work.

This subsection needs to explain if food availability was considered, in my interpretation it was not, and if not, why as it is a key distribution factor for any type of crab. Then, introduce the same food availability factor in the discussion (currently it is very incipient therein)

**Response：**Thank you for your valuable feedback. In response to your comments, although food availability was not directly considered, we have included environmental factors such as chlorophyll, nutrients, primary productivity, and organic carbon in our study (Table 1), which serve as indirect indicators of food supply. These factors are closely related to the food resources available to horseshoe crabs and thus can reflect the influence of food availability on their distribution. Furthermore, we have added a detailed explanation regarding food availability in the discussion section, particularly in Lines 472-475, to address this important aspect of their distribution. We believe these revisions have strengthened the manuscript and provided a more comprehensive understanding of the factors influencing horseshoe crab distribution. Thank you again for your helpful suggestions.

This text should be deleted as: 1. its content is more suitable in the introduction section and, 2. it' already given in previous sections. the Discussion should start with interpretation and contextualisation of main results.

**Response：**Thank you for your valuable feedback. We have deleted the text as requested.

this mechanism needs further clarification on how food can vary with depth and known crab responses to food availability

**Response：**Thank you for your suggestions. We have added further explanations on how food varies with depth and known horseshoe crabs responses to food availability as requested. The specific additions are as follows:

Water depth affects the distribution and abundance of horseshoe crab food sources such as plankton and benthic organisms. For example, in shallow water areas with sufficient light, the growth of phytoplankton is promoted, which in turn provides food sources for benthic organisms and indirectly affects the food supply of horseshoe crabs. Additionally, horseshoe crabs migrate annually in response to seasonal and water temperature changes. Every November, they swim from shallow seas to deep-water areas for overwintering; the following April to May, they return to shallow seas for reproductive migration. During migration, horseshoe crabs move between regions with different water depths and obtain different food sources. For example, while overwintering in deep-water areas, they may feed on certain deep-sea organisms; after returning to shallow seas, their food sources change again.

The paragraphs are too long and very difficult to follow from the readers' point of view; create breaks when the topic changes such as here. apply this logic everywhere in the doc.

**Response：**Thank you for your valuable feedback. In response to your concern about the lengthy paragraphs being difficult to follow, I have carefully revised the text by dividing the original paragraph into four thematic sections to enhance clarity and readability (Line 437-522).

This is a key discussion point that requires much more explanation for the entire study. in fact, the methods do not fully clarify if the data obtained in databases corresponds to adults or larvae and what are the implications of it. as stated here, larvae are likely to have different environmental requirements from adults. in other words, are the results being interpreted as simultaneously applicable to larvae and adults? if yes, this needs further justification as it represents a key point for conservation efforts, which are claimed to be supported by this study.

**Response：**Thank you for your feedback. In reality, the observational data in the database primarily pertains to adult horseshoe crabs, as adults are more easily observable. In fact, based on the distribution points (water depths) we analyzed, it is clear that the data used mainly represents the distribution of adult horseshoe crabs. Their ecological niche is broader than that of juveniles. We have clarified in the Materials and Methods section (Line 149) that the data used pertains to adult horseshoe crabs.

all this is an hypothetical argument, please provide references for support and clarify how wind at surface can affect the behaviour of crabs at the bottom, particularly further away from the shore..

**Response：**Thank you for your feedback. We have added supporting literature and a new paragraph to further clarify the impact of wind on benthic crabs.

The added paragraph reads: "Wind exerts a profound influence on the survival of marine benthic organisms. Notably, short-term extreme storms can readily induce stranding of marine benthic organisms [48]. For instance, in the aftermath of Storm Emma (February 28 to March 5, 2018), a large-scale stranding event involving various benthic organisms occurred on Punta Umbria Beach in Spain [48]. Following long-term adaptive evolution, wind speed has come to shape the composition and distribution patterns of benthic communities."(Line 477-483)

The added reference is: García-de-Lomas J, Payo A, Cuesta JA, Macías D. Morphodynamic Study of a 2018 Mass-Stranding Event at Punta Umbria Beach (Spain): Effect of Atlantic Storm Emma on Benthic Marine Organisms. JMSE. 2019;7: 344. doi:10.3390/jmse7100344.

These additions provide empirical evidence and further theoretical explanation to support our arguments regarding the impact of wind on benthic crabs, addressing the reviewer's concerns.

This result presentation is too long and extensively repeats that of the results section. summarise and go straight to their interpretation.

**Response：**Thank you for your constructive feedback regarding the length and redundancy of the result presentation. We have taken your comments into consideration and revised the section accordingly (Line 532-536).

This entire subsection is unwarranted and should be removed or very summarised into a subsection dedicated to relate the study results into tentative clear recommendations for habitat conservation. There is no clear use of this info for the reader when trying to understand the contribute of this work to the conservation of the crabs (as claimed since the abstract!).

**Response：**Thank you for your constructive feedback. We have carefully considered your suggestion and agree that the subsection in question does not contribute significantly to the main focus of the manuscript. Therefore, we have removed the entire subsection as recommended.

this is old news since the intro, not relevant at this stage of the manuscript

**Response：**Thank you for your constructive feedback. We have removed this section based on your previous suggestion.

This section needs total re-drafting, changing from yet another summary of results, to the impacts, contributions and applications of the new knowledge generated.

**Response：**Thank you for your constructive feedback. We have carefully considered your suggestion, as well as the advice from another reviewer, and have revised the section accordingly. The section has been completely re-drafted to focus on the impacts, contributions, and applications of the new knowledge generated, rather than simply summarizing the results. We believe this revision strengthens the manuscript and provides a more comprehensive discussion of the implications of our findings (Line 682-707).

**Reviewer #2:** The study presents a robust analysis of the distribution and habitat characteristics of Asian horseshoe crabs (Tachypleus tridentatus, Tachypleus gigas, and Carcinoscorpius rotundicauda), employing predictive modeling to infer both historical and contemporary patterns. The use of the MaxEnt model and niche overlap metrics are well-established and appropriate methodological approaches for the study's objectives.

Despite its scientific relevance and potential impact on species conservation, the manuscript has some weaknesses, including: 1) a lack of deeper justifications for certain methodological choices; 2) some model-based inferences that require stronger support from the literature; and 3) minor textual adjustments to improve clarity and argumentation.

More information about suggestions the authors can to access in the PDF archive annex to this review.

Thus, I recommend that the manuscript undergo revision before acceptance for publication, incorporating the suggested adjustments outlined below.

CONTRIBUTION TO AUTHORS

SUGGESTION FOR TITLE

In my view, the title "Exploring habitat characteristics, distribution, and niche relationships of Asian horseshoe crabs: preserving living fossils and biodiversity" is relatively long and could be more focused on the main topics addressed. Therefore, I suggest the title "Modeling habitat distribution and niche overlap of Asian horseshoe crabs: glacial refugia and conservation priorities" which incorporates niche modeling and overlap, as well as the significance of glacial refugia for conservation.

**Response：**We sincerely appreciate your valuable suggestions for optimizing the paper's title. We fully concur with your assessment that the original title was lengthy and lacked focus. However, following the third reviewer's recommendation, we have removed references to "glacial refugia." Based on your feedback, we have revised the title to: " Modeling habitat distribution and niche overlap of Asian horseshoe crabs: Implications for Conservation"

ABSTRACT

The abstract is well-structured but could be improved, particularly by clarifying the implications of the results and strengthening the connection between the findings and conservation. Thus, I suggest structuring the abstract as follows “Asian horseshoe crabs are ancient organisms essential for the balance of marine ecosystems. However, detailed information on their ecology and the environmental factors influencing their distribution remains limited. In this study, we analyzed habitat characteristics, potential distribution, niche overlap, and refugia during the Last Glacial Maximum (LGM) for three species: Tachypleus tridentatus, Carcinoscorpius rotundicauda, and Tachypleus gigas. Predictive modeling using MaxEnt and niche analysis revealed that water depth and proximity to land are key factors determining species distribution, with species-specific environmental influences: T. tridentatus is affected by maximum summer chlorophyll-a, C. rotundicauda by minimum chlorophyll-a, and T. gigas by wind velocity. Niche overlap was highest between C. rotundicauda and T. gigas, while T. tridentatus showed lower overlap with the other species. During the LGM, refugia for T. tridentatus were concentrated in the Minjiang and Pearl River estuaries, while C. rotundicauda and T. gigas found shelter in the Malacca Strait region. The results highlight priority conservation areas, providing insights for management and protection strategies amid current environmental threats.”

**Response：**Thank you sincerely for your meticulous review of the abstract and your constructive feedback. We fully agree with your assessment that the abstract could benefit from further strengthening the connection between the findings and their conservation implications. Following your suggestions, as well as those from the other two reviewers, we have revised the abstract to more clearly present the core discoveries and their ecological conservation significance. Please find below the revised version of the abstract:

Asian horseshoe crabs are ancient organisms essential for the balance of marine ecosystems. However, detailed information on their ecology and the environmental factors influencing their distribution remains limited. In this study, we analyzed habitat characteristics, potential distribution, and niche overlap for three species: *Tachypleus tridentatus*, *Carcinoscorpius rotundicauda*, and *Tachypleus gigas*. Predictive modeling using MaxEnt and niche analysis revealed that water depth and distance to land are key factors determining species distribution, with species-specific environmental influences: *T. tridentatus* is affected by maximum summer chlorophyll-a, *C. rotundicauda* by minimum chlorophyll-a, and T. gigas by wind speed. In terms of niche overlap, the highest degree of overlap was observed between *C. rotundicauda* and *T. gigas*, while the overlap between *T. tridentatus* and T. gigas was the lowest. The results highlight priority conservation areas, providing insights for management and protection strategies amid current environmental threats.

INTRODUCTION

The introduction has a solid and well-founded structure, contextualizing the importance of Asian horseshoe crabs, their threats, and the need for predictive modeling in conservation. However, it can be improved in terms of fluency, reduction of redundancies, and making the text more direct and impactful. The main suggested changes are as follows:

Page 2, Lines 45-54: The idea that horseshoe crabs are "living fossils" and that their morphology has remained unchanged for millions of years is repeated. This information is already mentioned earlier in the introduction. Therefore, I suggest a more concise mention to avoid redundancy: "Horseshoe crabs, whose morphology has remained virtually unchanged for over 200 million years, play a fundamental role in marine ecosystems and biodiversity maintenance."

**Response：**Thank you sincerely for your meticulous review of the introduction section and your valuable suggestions. We fully agree with your recognition of the introduction's structure and also deeply understand the importance of enhancing text fluency, reducing redundancies, and increasing directness and impact. In response to your revision suggestions, we have carefully reviewed them and made optimized adjustments to the relevant content based on your recommendations.

Regarding the repeated description on Page 2, Lines 45-54 about horseshoe crabs as "living fossils" and their unchanged morphology over millions of years, we have adopted your suggestion and streamlined it. The revised statement is as follows: " Horseshoe crabs, as ancient creatures, have fossils dating back to the Ordovician period of the Palaeozoic Era approximately 475 million years ago [1–3]. Their morphology has remained virtually unchanged for over 200 million years, playing a fundamental role in marine ecosystems and the maintenance of biodiversity [4,5]." This phrasing retains the key information while avoiding redundancy, making the text more concise and impactful.

Page 3, Lines 60-68: The relevance of horseshoe crabs in marine ecology is reiterated similarly to previous statements. This should either be removed or reformulated to highlight new aspects of their ecological function, such as their interactions with food chains or environmental vulnerability.

**Response：**Thank you for your attentive feedback. The previous text presents a general overview of the ecological significance of horseshoe crabs, with lines 60-80 focusing on the geographic distributions and specific habitat characteristics of the four global species. We have implemented targeted revisions to reduce content redundancy while maintaining informational integrity.

Page 3, Lines 77-88: The transition between threats and predictive modeling is abrupt. The text shifts from habitat degradation directly to MaxEnt modeling without a clear connection. I suggest adding a transition sentence explaining how modeling can aid in mitigating environmental impacts and conserving the species: "Given habitat degradation and the need to identify priority conservation areas, species distribution models become essential tools. MaxEnt, widely used in ecological studies, enables highly accurate predictions of species distribution."

**Response：**Thank you for your constructive feedback. Indeed, following our elaboration on habitat degradation of Asian horseshoe crabs, we included a concluding sentence at the end of the paragraph to emphasize the urgent need for in-depth research: "Therefore, conducting in-depth research on the potential habitats of the three Asian horseshoe crab species (*T. tridentatus*, *C. rotundicauda*, and *T. gigas*) and the important ecological factors affecting their distribution is crucial for formulating scientific and reasonable conservation strategies, promoting population recovery, and protecting biodiversity." This sentence effectively transitions to the subsequent section. Prior to introducing MaxEnt, we explicitly stated how species distribution models (SDMs) can address these research gaps: "Species distribution models are an effective method to help researchers gain a deeper understanding of the intricate relationship between species and their environments." This logical flow ensures smooth connectivity between the two paragraphs, first highlighting the problem and then presenting the methodological solution.

Page 4, Lines 105-113: The study objectives are presented, but no mention of a testable hypothesis is made. I suggest adding: "We hypothesize that water depth, land distance, and specific environmental factors are the primary determinants of the distribution of the three Asian horseshoe crab species. We use the MaxEnt model to predict potential habitats and analyze niche overlap, aiming to provide insights for species conservation."

**Response：**Thank you for your valuable suggestions to this research. We fully acknowledge the importance of clearly stating testable hypotheses in the study. In response to your comment regarding the lack of a testable hypothesis on page 4, lines 105-113, we have refined our study design and now include the following supplemental hypothesis:

As horseshoe crabs are typical intertidal benthic organisms, we hypothesize that water depth, land distance, and specific environmental factors are the primary determinants of the distribution of the three Asian horseshoe crab species. We use the MaxEnt model to predict potential habitats and analyze niche overlap, aiming to provide insights for species conservation.

MATERIALS AND METHODS

This section is well-structured, adequately detailing data collection, predictive modeling, and statistical analysis. The inclusion of Table 1, which presents important environmental variables and their corresponding Variance Inflation Factor (VIF) values for the three species, is excellent. However, I suggest a few improvements:

Page 5, Lines 120-130: The text does not mention how potential errors in occurrence records, such as inaccurate or outdated coordinates, were handled. I suggest adding: "In addition to removing duplicates and anomalous points, records with inaccurate coordinates or located in terrestrial environments were manually verified and corrected whenever possible."

**Response：**Thank you for your meticulous review of our research methodology. Regarding data preprocessing, we indeed first removed duplicates and outliers, followed by manual verification and correction of records with inaccurate coordinates or those located in terrestrial environments. The manuscript has now been revised as per your requirements.

Page 8, Lines 153-155: The arbitrary criterion for splitting training (75%) and testing (25%) data is not justified. It would be important to insert this justification, such as: "The 75/25 (training/testing) split was chosen as it is widely used in ecological modeling studies, ensuring a balance between adequate model training and robust validation."

**Response：**Thank you for your valuable suggestion regarding the rationale for data splitting. We have revised the manuscript according to your advice, replacing "To ensure prediction accuracy, the process involved randomly selecting 25% of the distribution points as the test set, while the remaining 75% served as the training set for model calibration and testing." with " The 75/25 (training/testing) split was chosen as it is widely used in ecological modeling studies, ensuring a balance between adequate model training and robust validation.".

Page 8, Lines 155-157: The text states that the MaxEnt model was run until convergence but does not mention whether adjustments for regularization, maximum iterations, pseudo-absence numbers, or other key parameters were made. Therefore, I suggest including: "MaxEnt parameters were adjusted to minimize overfitting, using a regularization value of X, a maximum iteration number of Y, and 10,000 randomly generated pseudo-absences within the study area."

**Response：**Thank you for your constructive feedback regarding the MaxEnt model parameterization. We have now added " MaxEnt parameters were adjusted to minimize overfitting, using a regularization value of 1, a maximum iteration number of 5000, and 10,000 randomly generated pseudo-absences within the study area." as requested, which specifies the regularization value (X = 1) and maximum iteration number (Y = 5000) used in our analysis.

Page 8, Lines 172-176: It would be important to explain why the Schoener index was used to measure niche overlap. I suggest adding: "The Schoener index (D) was chosen as it is widely used in ecological niche studies, providing a robust measure of niche overlap by considering both presence differences and habitat suitability intensity."

**Response：**Thank you for your constructive feedback regarding the niche overlap analysis. We have now incorporated "Sentence A" into the manuscript as you suggested.

RESULTS

The Results section provides detailed and consistent information on the habitat modeling of the three Xiphosura species, with excellent organization and statistical robustness. However, some improvements can enhance clarity, avoid redundancies, and strengthen data interpretation. Below are some suggestions for improvement:

Page 9, Lines 184-189: AUC values are repeatedly presented for each species, which could be synthesized into a table for easier reading. Therefore, I suggest including the following text and table: "AUC values were above 0.99 for all species (T. tridentatus: 0.9963/0.9958, C. rotundicauda: 0.9980/0.9972, T. gigas: 0.9974/0.9972), indicating high model reliability (Table ??)."

**Response：**Thank you for your constructive feedback regarding the presentation of AUC values in the Results section. We agree that synthesizing these values into a table would enhance readability and have implemented this suggestion as follows: " After ROC curve analysis, the AUC values for the training and testing sets were above 0.99 for all species (T. tridentatus: 0.9963/0.9958, C. rotundicauda: 0.9980/0.9972, T. gigas: 0.9974/0.9972), indicating high model reliability (Table 2)."

We have created Table 2 to present the AUC values in a clear and organized manner, which we believe improves the flow and readability of the Results section. The table includes AUC values for both training and testing datasets, providing a comprehensive overview of model performance across species.

Page 9, Lines 194-196: The text lists key variables but does not explain their ecological importance for each species. I suggest including: "Water depth may be related to the availability of spawning substrate, while chlorophyll-a reflects primary productivity, influencing food availability. Wind velocity may affect larval dispersal and sediment transport."

**Response：**Thank you for your insightful feedback regarding the ecological importance of key variables. We have incorporated your suggestions and added the following text after the sentence "The results of the MaxEnt model analysis revealed the environmental variables that most significantly shape the current potential habitats of the three Asian horseshoe crab species.":

"These variables have crucial impacts on horseshoe crab distribution; for instance, water depth may be related to the availability of spawning substrate, while chlorophyll-a reflects primary productivity, influencing food availability. Wind velocity may affect larval dispersal and sediment transport."

Page 12, Lines 273-284: The D coefficient is presented, but its implication for species coexistence (biological significance of niche overlap) could be improved. Suggested revision: "The low overlap between T. tridentatus and T. gigas suggests ecological segregation, possibly due to distinct habitat and dietary preferences. In contrast, the high overlap between C. rotundicauda and T. gigas may indicate stronger competition for resources, requiring additional niche differentiation mechanisms."

**Response：**Thank you for your valuable feedback on improving the biological significance of niche overlap as it pertains to species coexistence. We have made revisions in line with your suggestions, specifically adding the following text on Line 353-356: "The low overlap between *T. tridentatus* and *T. gigas* suggests ecological segregation, possibly due to distinct habitat and dietary preferences. In contrast, the high overlap between *C. rotundicauda* and *T. gigas* may indicate stronger competition for resources, requiring additional niche differentiation mechanisms." We appreciate your guidance and believe these changes enhance the clarity and depth of our analysis.

Page 13, Lines 298-303: The study demonstrates that species had distinct refugia during the LGM but does not discuss how this may influence conservation strategies. Suggested addition: "The identification of refugia suggests that these areas may harbor genetically resilient populations, making them conservation priorities."

**Response：**Thank you for your valuable suggestions on enhancing the impact of refugia research on conservation strategies during the LGM. In accordance with the third reviewer's recommendation, we have removed the results and discussion pertaining to the LGM period. Thank you again for your advice.

FIGURES

The figures effectively visualize spatial distribution patterns and ecological niches of the species and are well-structured. Below are some improvements to enhance clarity and accessibility of the data:

Figure 1: Recommend labeling key geographic regions mentioned in the text, such as the Malacca Strait and the Gulf of Thailand.

**Response：**Thank you for your valuable suggestions regarding Figure 1. We have labeled the key geographic regions in Figure 1: the Gulf of Thailand and the Malacca Strait.

Figure 2: Each species’ variables could be represented by distinct colors (lighter shades), with the three most important variables for each species highlighted in more contrasting (darker) colors. Another suggestion is to place the species' names directly on each graph instead of using a legend (red square followed by species name).

**Response：**Thank you for your constructive feedback on Figure 2. We have implemented your suggestions to enhance the clarity and interpretability of the graph. Firstly, we have assigned distinct color schemes to each species: *T. tridentatus* is represented by red and dark red, *C. rotundicauda* by blue and dark blue, *T. gigas* by green and dark green. Within each species' color scheme, the three most important variables are highlighted in the deeper shade (dark red, dark blue, dark green) to emphasize their significance, while the remaining variables are shown in the lighter shade. Additionally, we have placed the species' names directly on each graph, as you recommended, to improve readability and eliminate the need for a separate legend. We appreciate your guidance and believe these revisions will make Figure 2 more informative and user-friendly.

Figure 3 (A, B, and C): Legends could be more intuitive by using a more perceptible color gradient (e.g., yellow for low suitability, orange for moderate suitability, and red for high suitability). Additionally, indicating key ecological areas directly on the maps would facilitate more direct access to information.

**Response：**Thank you for your valuable suggestions on improving Figure 3 (A, B, and C). We have carefully considered your recommendations and made the following revisions: Regarding the color gradient for suitability levels, we agree that using a more perceptible gradient is essential for clarity. However, since the land areas are already depicted in earthy yellow tones, we decided to use blue, light red, and dark red to represent low, moderate, and high suitability in marine areas, respectively. This choice avoids potential confusion that might arise from using yellow and orange in both terrestrial and marine contexts. Additionally, we have indicated key ecological areas directly on the maps, as you suggested, to facilitate more direct access to information. Specifically, we have marked important geographic regions such as the Gulf of Thailand, the Indochina Peninsula, Sumatra, Borneo, and Java. We appreciate your guidance and believe these revisions enhance the interpretability and usefulness of Figure 3.

Figure 4: Clarity could be improved by standardizing the axes for the same relationships, allowing direct comparison between the three species, with points colored as in Figure 2. This would enable comparisons of the three species for each column, placing titles above the left column—Depth (m)—and the middle column—Land Distance (km)—which would compose Figure 4 (letters A, B, D, E, G, and H). Chlorophyll-a (Maximum or Summer ...) would form another figure, while Wind Velocity (m/s) would be isolated as another figure. This restructuring could enhance readability. Alternatively, all graphs could remain in Figure 4 but grouped within frames labeled with the variable name. Two vertical columns with three graphs each (left and center) and two graphs (chlorophyll-a) with another for wind velocity would be displayed. Maintaining the same axis values for comparable variables is crucial, with distinct color points representing each species (as suggested for Figure 2).

**Response：**Thank you for your suggestions. I attempted to redraw the figure following your advice, but the result was very unsatisfactory. This was mainly due to the excessive number of points in the figure. Although three colors were used to distinguish them, the points of the later-added species overlapped with those of the previously-added ones, resulting in a very unclear image. Therefore, we suggest retaining the original figure. However, to make it clearer, we have added the Latin names of the species to each figure, which facilitates comparative analysis. Thank you again for your detailed comments.

Figure 5: Could include a graphical representation of niche distribution in environmental space for better interpretation. Additionally, a bar graph illustrating absolute niche overlap values would be beneficial.

**Response：**We sincerely appreciate your constructive suggestions regarding Figure 5. After careful consideration, we have two primary concerns about implementing the proposed revisions: **(1)** Niche Distribution Representation: While we recognize the potential value of visualizing niche distribution in environmental space, this information is already comprehensively addressed in our habitat suitability modeling presented in Section 3.2 **Potential habitats of *T. tridentatus*, *C. rotundicauda*, and *T. gigas***. The current Figure 4 explicitly maps predicted habitat distributions across the environmental gradient, which we believe sufficiently conveys spatial niche patterns. Adding another graphical layer here may create redundancy rather than enhance interpretation. **(2)** Niche Overlap Bar Graph: Due to methodological constraints related to species occurrence data quality, our pairwise niche overlap analysis yielded only three comparable values. A bar graph with three data points would visually underperform compared to alternative presentations. Instead, we elected to directly label these values on the niche overlap graphs (Figures 5A, 5C, 5E) as this presentation is more conspicuous and facilitates interpretation.

Thank you again for your insightful feedback - we value this opportunity to strengthen our manuscript.

Figure 6 (A, B, and C): The obtained results do not allow for arrows indicating possible post-LGM species dispersal routes. Another relevant aspect would be overlaying current distributions to facilitate past and present comparisons.

**Response：**We appreciate your constructive feedback on Figure 6. In response to Reviewer 3's earlier comments, we have removed all Last Glacial Maximum (LGM)-specific results and discussions to avoid over-interpretation of paleodistribution models, which represent only one lineage and have limited impact on informing horseshoe crab conservation strategies.

DISCUSSION

The Discussion section is well-structured, addressing the study's main findings and relating them to previous research. However, some aspects could be improved to make the text more concise, enhance its flow, and strengthen the connection between the results and species conservation.

Page 14, Line 353 and Page 15, Line 358-364: The text mentions the importance of depth and chlorophyll-a but does not detail how these factors directly influence horseshoe crab behavior and survival. Suggested addition: "Depth influences not only the availability of suitable spawning substrate but also access to food sources and protection from predators. Similarly, chlorophyll-a reflects primary productivity, impacting the abundance of organisms forming the horseshoe crab food base."

**Response：**We sincerely appreciate your valuable suggestions. In the discussion section titled "Key environmental factors influencing the distribution of three species of Asian horseshoe crabs," we have elaborated on the specific mechanisms through which key environmental factors, such as water depth and chlorophyll-a concentration, influence the behavior and survival of the three Asian horseshoe crab species. For instance, we have analyzed how water depth affects the horseshoe crabs' selection of spawning substrates, access to food resources, and ability to avoid predators. Concurrently, we have also explored how chlorophyll-a concentration, as an indicator of primary productivity, reflects the abundance of food sources for horseshoe crabs, thereby influencing their survival strategies. Furthermore, in response to the reviewer's suggestion, we have added a concluding statement at the end of this paragraph to further emphasize the significance of these environmental factors. The specific addition is as follows: "In summary, depth influences not only the availability of suitable spawning substrate but also access to food sources and protection from predators. Similarly, chlorophyll-a reflects primary productivity, impacting the abundance of organisms forming the horseshoe crab food base."

These revisions aim to provide a more comprehensive overview of the integrated impacts of environmental factors on the survival and distribution of Asian horseshoe crabs and ensure a more in-depth and complete analysis.

Page 15, Lines 367-376: The study presents niche overlap coefficients among species but could better explore the conservation and management implications of these results. Suggested addition: "The high overlap between C. rotundicauda and T. gigas may indicate greater competitive pressure, requiring additional strategies for habitat differentiation or temporal reproductive shifts to reduce ecological conflicts. This information can inform management policies to prevent degradation of critical shared habitats."

**Response：**Thank you for your valuable suggestions. We appreciate your insights regarding the niche overlap and conservation implications among the three Asian horseshoe crabs. We would like to kindly point out that the concerns you raised have already been addressed in the Discussion section of our manuscript, specifically in the subsection titled "Niche overlap and resource utilization of three Asian horseshoe crabs". As highlighted in the last sentence of that paragraph:

"However, it may also prompt them to develop more refined differentiation strategies during ecological adaptation and evolution, such as temporal niche partitioning and fine-grained division of food resources. These strategies are crucial for maintaining population stability and ecological balance."

This sentence directly echoes your recommendation to discuss the potential for species to evolve strategies that mitigate competition, such as temporal reproductive shifts and habitat differentiation. We believe that this existing text in our manuscript already captures the essence of your suggestions and provides a solid foundation for further exploration of conservation and management implications.

Thank you again for your constructive feedback. We are confident that our manuscript, as currently presented, thoroughly addresses the niche overlap and its ecological implications for the three Asian horseshoe crabs.

Page 17, Lines 424-447: Identifying refugia during the LGM is an important aspect of the study, but the text does not explore how this information can be useful for current conservation. I strongly recommend expanding this section. One possible addition: "The identification of historical refugia suggests areas that potentially harbor genetically resilient populations. Protecting these regions may be crucial for ensuring species population viability in future climate change scenarios."

**Response：**We appreciate your insightful suggestion regarding the potential conservation implications of identifying refugia during the LGM. However, in response to another reviewer's recommendation, we have removed all results and discussion pertaining to the LGM period from the manuscript. This decision was made to streamline the focus of the study and address other key aspects highlighted by the reviewers. Consequently, the section you recommended expanding is no longer present in the revised version of the manuscript. We thank you for your understanding and hope that the revised manuscript meets your expectations in other aspects.

Page 18, Lines 450-463: The discussion assertively lists threats to Asian Xiphosura species, such as habitat destruction and overfishing, but does not suggest concrete measures to mitigate these impacts. It would be expected for the authors to mention this. Suggested addition: "The implementation of marine protected areas and monitoring programs is recommended to assess population responses to environmental impacts. Furthermore, regulating the fishing and biomedical harvesting of horseshoe crabs should be strengthened to ensure a balance between conservation and the sustainable use of this biological resource."

**Response：**We appreciate the reviewer's valuable criticism regarding the lack of specific mitigation measures in our discussion of threats to Asian Xiphosura species. Although our manuscript highlights pressing issues such as habitat destruction and overfishing, we recognize the importance of proposing feasible solutions. In response to the reviewer's suggestions, we have added: "To effectively address the pressing conservation challenges outlined above and mitigate the threats to Asian horseshoe crabs, it is imperative to implement a combination of protective measures and sustainable management strategies. The implementation of marine protected areas and monitoring programs is recommended to assess population responses to environmental impacts. Furthermore, regulating the fishing and biomedical harvesting of horseshoe crabs should be strengthened to ensure a balance between conservation and the sustainable use of this biological resource."

CONCLUSIONS

The conclusions could be substantially improved by a revision to avoid repetitions (e.g., repeating environmental factors such as chlorophyll-a and depth as influential factors) and to make the text more direct. Additionally, concrete conservation policy recommendations could be included (e.g., proposing the implementation of marine protected zones in areas identified as critical habitats and historical refuges, along with strengthening regulations on the harvest of these species for biomedical purposes, aiming for a balance between conservation and sustainable use). Moreover, the connection between historical distribution and future climate change could be emphasized (e.g., future analyses to assess the effects of global warming and ocean acidification on the distribution of horseshoe crabs, potentially shifting their habitats to new areas). Finally, the importance of MaxEnt modeling as a predictive tool for conservation should be highlighted (e.g., MaxEnt modeling identified habitat distribution patterns and provided key insights for conservation using predictive data, representing an advancement in the ecological understanding of these species).

**Response：**We appreciate the reviewer's constructive feedback and have revised the conclusions section to address the points raised. The text has been streamlined to eliminate redundancies, such as repetitive mentions of environmental factors like chlorophyll-a and depth, and made more direct.

Revised Conclusions：

This study offers a comprehensive analysis of the habitat characteristics, current potential habitat distributions with varying suitability levels, and niche overlap and similarity among three Asian horseshoe crab species: *T. tridentatus*, *C. rotundicauda*, and *T. gigas*. The findings reveal that water depth and distance to land are shared critical factors shaping the potential habitat distribution of these species. However, each species is also influenced by distinct environmental variables: *T. tridentatus*'s distribution is further limited by summer (May-Oct) maximum chlorophyll-a concentration, *C. rotundicauda*'s habitat is closely tied to minimum chlorophyll-a concentration, and *T. gigas*'s habitat selection is notably impacted by wind speed. In terms of niche overlap, *C. rotundicauda* and *T. gigas* show the highest overlap (Schoener's D = 0.62), signifying similar ecological resource use, whereas *T. tridentatus* and *T. gigas* exhibit the lowest overlap (Schoener's D = 0.22), indicating clear niche differentiation. This research provides crucial insights for the conservation and management of these evolutionarily important "living fossils." To enhance protection, we recommend establishing marine protected areas (MPAs) in identified critical habitats, particularly focusing on regions where habitat overlap is high, to safeguard shared ecological resources. Additionally, strengthening regulations on the harvest of these species for biomedical purposes is essential to balance conservation with sustainable use. Proactive strategies are needed to assess the impacts of global warming and ocean acidification on horseshoe crab distributions. This includes monitoring potential habitat shifts to new areas due to changing environmental conditions, such as alterations in chlorophyll-a concentrations and wind speed, to inform adaptive conservation measures. The study underscores the value of MaxEnt modeling as a predictive tool in conservation. By identifying habitat distribution patterns and offering critical insights through predictive data, MaxEnt modeling advances our ecological understanding of these species, aiding in the development of effective conservation strategies. Future applications of this model could simulate habitat changes under different climate scenarios, further supporting adaptive management decisions.

**Reviewer #3: Overall:**

This is overall a very good and interesting paper. The authors used publicly available datasets to model the spatial distribution, habitat suitability, and niche overlap among the three Asian species of horseshoe crabs, with the aim of identifying important habitats for conservation planning for these threatened or potentially threatened (data-deficient, understudied) species. Exercises were also done to identify the key environmental variables determining each species’ habitat distribution and their potential habitat and refugia during the Last Glacial Maximum (LGM). For the most part, the paper is well written, the figures are good, and the analyses are done and interpreted logically. I particularly liked how the authors went through the key variables determining habitat that they identified in the Discussion to assess why these should be important for the studied species. The study is significant, novel, and should be published after some revisions. I make some comments below about places where the paper might benefit from clarifications or additional Discussion points, but with one exception these are mostly minor concerns. My one larger concern relates to the LGM analysis, which (maybe more by brevity/omission of details than intended misdirection) may appear to the reader as an equivalent analysis to the main modelling efforts, when in reality it was a much more limited exercise with less certain results. As I elaborate below, there needs to be much more clarity on exactly how this was done, and probably also more caution in interpreting its results.

Please note: I consider that Minor Revisions are needed for the majority of the paper, although suggested changes to the details presented for the LGM analysis (or even its removal) as detailed below may be considered more extensive (Major).

Specific comments, roughly in order of their occurrence in the text:

(1) This is a very minor gripe, but it confused that the three horseshoe crab species’ names are listed in no apparent order for no clear reason in some parts of the paper (e.g., lines 129-130, 133-134, 215-216, 24-246). In other parts Tachypleus species are listed before C. rotundicauda, which at least has some logic (e.g., lines 55-6, 61-68, 85-86), although it is still a little strange to list T. tridentatus before T. gigas; this latter order does at least match the alphabetical order of the common names given in lines 54-56. I think it would be preferable to list the species’ names in actual alphabetical order at most/all places in the text – so Carcinoscorpius rotundicauda, Tachypleus gigas, and T. tridentatus. This would also apply to how the results are presented.

**Response：**We appreciate your constructive feedback. Indeed, in the introduction section, we listed the English names of the four globally distributed species in alphabetical order as you observed. However, in the subsequent sections, including Materials and Methods, Results, and Discussion, we arranged the three Asian horseshoe crabs based on their geographic distribution from north to south. While adhering to a strict alphabetical order, as you suggested, is a valid approach, we considered it more convenient for presenting results and discussions to maintain a consistent order based on their geographic distribution from north to south (i.e., *T. tridentatus*, *Carcinoscorpius rotundicauda*, and *Tachypleus gigas*). Therefore, we have revised the order of species presentation in the introduction to align with this geographic distribution for consistency throughout the entire manuscript.

(2) Lines 90-104, 149-153: MaxENT was used for species distribution modelling in this study. Nothing wrong with this, it is a well-established and widely used method. The authors do note around line 90 that other techniques are available, however, and it may be worth noting alternative approaches in the Discussion somewhere. Could this analysis be repeated (maybe in future) with different approaches, such as what, with what added benefits/drawbacks, and would the outcomes be expected to be very different, etc.?

**Response：**Thank you for your suggestion. We have added a section in the Discussion titled "The Selection of the Model and the Accuracy of the Results" (Line 394-426). In this section, we introduce several common species distribution models, detail their respective advantages and disadvantages, elaborate on the rationale behind choosing the MaxEnt model, and discuss the reliability of the results obtained using the MaxEnt model.

(3) Line 137: Just for clarity, should specify that this is “a resolution of 2.5 minutes latitude/longitude” (to avoid confusion with time).

**Response：**Thank you for your attentive suggestion. We have made the requested revision, changing "2.5 minutes" to "2.5 minutes of latitude/longitude" as advised.

(4) LGM Analysis: Lines 137-138: I think a lot of important detail is skipped over here.

The GMED database does include data layers for the Last Glacial Maximum (LGM), but as far as I can tell from browsing the website only a handful (4) of environmental variables are available there from the LGM – specifically water depth, sea surface temperature, salinity, and ice thickness. My understanding based on the paper is that the authors applied their habitat suitability/species distribution models for each of the three species derived for the present/historical period to the LGM data to determine past habitats and refugia (lines 293-315 in the Results, Fig. 6). However, key variables identified in the present-day models, such as chlorophyll-a and winds (lines 247-265), are not apparently available in the LGM data layers from GMED (indeed, of all the variables listed in Table 1, hardly any seem available in the LGM layers), so it is not clear to me how the authors could identify past habitats in this setup. This needs to be explained.

Maybe additional variables are available in GMED for the LGM that are not obvious on the public-facing webpage, but if so more information needs to be provided on where/how these were downloaded. A table like Table 1 showing which LGM data layers were considered would be useful regardless.

However, I suspect that maybe the present-day potential distributions were just applied to water depths and shorelines available during the LGM to identify past refugia, without considering other variables’ past values’ impact in habitat suitability. If so, not stating this clearly in the paper potentially misleads the reader. Further, there are important limitations to this approach that need to be addressed in the Methods and Discussion. For example, if chlorophyll concentration strongly impacts a species’ distribution, then can we be sure that an area with suitable chlorophyll levels today would have had suitable levels in the LGM, even if it was available habitat based on depth and shoreline? I think not. Consider that today’s mid-deeper water shelf seas would have been shallow coastal areas during the LGM, with correspondingly different currents, light levels, etc. impacting plankton productivity.

In the discussion using paleoenvironmental data (line 444) to better address this in the future is mentioned – is this because such data were not used in the present study? If so this is not obvious from the Methods and Results as written.

It might even be worth considering removing the LGM analysis entirely, given the potential problems identified above. I see it as a minor side analysis to the main part of this study, and do not think a lot of important material is lost by not identifying LGM refugia – the study still produces habitat suitability and niche overlap maps for the species and identifies important variables, which is most useful for conservation planning.

**Response：**Thank you for your meticulous comments. The study of the LGM distribution and refugia locations is indeed a minor side analysis to the main part of this study and has limited guiding significance for the conservation of current habitats. Therefore, in order to make the article more focused and directly relevant to the main theme, we have followed your advice and removed all content related to the LGM period from the manuscript.

(5) Line 298: change “highly and moderately suitability” to “highly and moderately suitable”

**Response：**Thank you for highlighting this error. We have revised "highly and moderately suitability" to "highly and moderately suitable" as suggested.

(6) Lines 245-265, 338, and elsewhere: The authors should consider not presenting depths as negative numbers in the paper. It is logical and probably necessary for depth have a negative value when running your models (particularly to make it apply to the LGM when depths might become positive die to lower sea levels), but it become confusing to read in the text – most of us are used to thinking of the sea at a given point being 40 m deep, not “-40 m deep”. Some readers may even misread the negative values to imply depths above the sea level, below the sea bed, at some point in between the surface and bottom (e.g., 40 m down), etc.

**Response：**We appreciate your thoughtful feedback regarding the presentation of depth values. We have revised all depth-related numerical values from negative to positive throughout the entire manuscript.

(7) Temperature is included in the modeling exercise as an environmental variable, but is not one of the main ones found to impact the habitat distributions of the studied species. This is somewhat surprising, given the widespread impacts of temperature on biota, particularly aquatic arthropods, but may well be true – the studied species are all found in and around the tropics mainly, so temperature may have less impact on their biology overall within their ranges, other than perhaps at the extremes. This still may be a point to raise in the Discussion, though, and can be tied in with potential conservation concerns related to climate change. Also, during the LGM, one wonders if temperatures might have been different enough to affect habitat suitability for these species? Maybe another Discussion point to add. SST data are available for the LGM from GMED, so there is potential to address this question in a future exercise.

**Response：**We sincerely appreciate your insightful observations regarding the role of temperature in our analysis. While temperature was not identified as a key factor influencing the distribution of the three Asian horseshoe crab species in our study—as we focused on the three most critical environmental variables for detailed analysis—this does not diminish its measurable influence. For instance, Figure 2 demonstrates that both minimum temperature and summer temperature (May-Oct) also affect their distribution to some extent. Accordingly, we will revise the Discussion section to include a detailed examination of temperature's role within the subsection titled "Key environmental factors influencing the distribution of three species of Asian horseshoe crabs." This expansion will clarify how temperature interacts with other variables in shaping habitat suitability, even when it does not emerge as a dominant driver in the models. Regarding the LGM section, we have already removed this content from the previous response, as requested in your earlier feedback. Thank you again for highlighting this important nuance—we agree that a nuanced discussion of temperature's context-dependent impacts will strengthen the manuscript's scientific and conservation relevance.
